# Predicting HPV association using deep learning and regular H&E stains allows granular stratification of oropharyngeal cancer patients

**DOI:** 10.1038/s41746-023-00901-z

**Published:** 2023-08-19

**Authors:** Sebastian Klein, Nora Wuerdemann, Imke Demers, Christopher Kopp, Jennifer Quantius, Arthur Charpentier, Yuri Tolkach, Klaus Brinker, Shachi Jenny Sharma, Julie George, Jochen Hess, Fabian Stögbauer, Martin Lacko, Marijn Struijlaart, Mari F.C.M. van den Hout, Steffen Wagner, Claus Wittekindt, Christine Langer, Christoph Arens, Reinhard Buettner, Alexander Quaas, Hans Christian Reinhardt, Ernst-Jan Speel, Jens Peter Klussmann

**Affiliations:** 1 Department of Hematology and Stem Cell Transplantation, University Duisburg-Essen, University Hospital Essen, Essen, Germany; 2 Institute of Pathology, Medical Faculty, University Hospital Cologne, Cologne, Germany; 3 Department of Otorhinolaryngology, Head and Neck Surgery, Medical Faculty, University Hospital Cologne, Cologne, Germany; 4 Center for Molecular Medicine Cologne (CMMC), University of Cologne, Medical Faculty, Cologne, Germany; 5 Department of Pathology, GROW – School for Oncology and Reproduction, Maastricht University Medical Center, Maastricht, The Netherlands; 6 Hamm-Lippstadt University of Applied Sciences, Hamm, Germany; 7 Department of Translational Genomics, University of Cologne, Faculty of Medicine and University Hospital Cologne, Cologne, Germany; 8 Department of Otolaryngology, Head and Neck Surgery, University Hospital Heidelberg, and German Cancer Research Center (DKFZ), Heidelberg, Germany; 9 Tissue Bank of the National Center for Tumor Diseases (NCT) Heidelberg, Germany, and Institute of Pathology, Heidelberg University Hospital, Heidelberg, Germany; 10 Department of Otorhinolaryngology and Head and Neck Surgery, GROW-School for Oncology and Reproduction, Maastricht University Medical Center, Maastricht, The Netherlands; 11 Department of Pathology, GROW-School for Oncology and Reproduction, Maastricht University Medical Center, Maastricht, The Netherlands; 12 Department of Otorhinolaryngology, Head and Neck Surgery, University of Giessen, Giessen, Germany; 13 Clinic of Otorhinolaryngology, Head and Neck Surgery, Klinikum Dortmund, University of Witten/Herdecke, Faculty for Health, Department of Human Medicine, Witten, Germany; 14 West German Cancer Center, University Hospital Essen, Essen, Germany; 15 German Cancer Consortium (DKTK), Heidelberg, Germany; 16 Present Address: Institute of Pathology, School of Medicine, Technical University of Munich (TUM), Munich, Germany

**Keywords:** Prognostic markers, Oral cancer, Cancer imaging

## Abstract

Human Papilloma Virus (HPV)-associated oropharyngeal squamous cell cancer (OPSCC) represents an OPSCC subgroup with an overall good prognosis with a rising incidence in Western countries. Multiple lines of evidence suggest that HPV-associated tumors are not a homogeneous tumor entity, underlining the need for accurate prognostic biomarkers. In this retrospective, multi-institutional study involving 906 patients from four centers and one database, we developed a deep learning algorithm (OPSCCnet), to analyze standard H&E stains for the calculation of a patient-level score associated with prognosis, comparing it to combined HPV-DNA and p16-status. When comparing OPSCCnet to HPV-status, the algorithm showed a good overall performance with a mean area under the receiver operator curve (AUROC) = 0.83 (95% CI = 0.77-0.9) for the test cohort (*n* = 639), which could be increased to AUROC = 0.88 by filtering cases using a fixed threshold on the variance of the probability of the HPV-positive class - a potential surrogate marker of HPV-heterogeneity. OPSCCnet could be used as a screening tool, outperforming gold standard HPV testing (OPSCCnet: five-year survival rate: 96% [95% CI = 90–100%]; HPV testing: five-year survival rate: 80% [95% CI = 71–90%]). This could be confirmed using a multivariate analysis of a three-tier threshold (OPSCCnet: high HR = 0.15 [95% CI = 0.05–0.44], intermediate HR = 0.58 [95% CI = 0.34–0.98] *p* = 0.043, Cox proportional hazards model, *n* = 211; HPV testing: HR = 0.29 [95% CI = 0.15–0.54] *p* < 0.001, Cox proportional hazards model, *n* = 211). Collectively, our findings indicate that by analyzing standard gigapixel hematoxylin and eosin (H&E) histological whole-slide images, OPSCCnet demonstrated superior performance over p16/HPV-DNA testing in various clinical scenarios, particularly in accurately stratifying these patients.

## Introduction

The incidence of human papillomavirus (HPV)-associated oropharyngeal squamous cell carcinomas (OPSCC), a cancer localized primarily at the tonsils or base of tongue, and to a lesser extent at the soft palate and the uvula – is rising in Western countries^1^. The distinct biology of this disease is appreciated by incorporating p16 status, as a surrogate marker for HPV-infection, to the most recent (8th) staging system of the American Joint Committee on Cancer – highlighting that HPV-associated OPSCC are distinct from their HPV-negative counterparts^2^.

Due to the better prognosis of HPV-positive tumors and the considerable side effects of multimodal therapies, attempts have been made to de-intensify therapy. Here, various strategies have been pursued, such as trans-oral surgery, reduction of radiotherapy or omission of chemotherapy in several ongoing clinical trials^1^. Decisive for de-escalation strategies is exact patient selection. Most often, only p16 is used as an inclusive biomarker. However, there are approximately 10–15% of cases which are discordant by p16 and HPV DNA- or RNA-status. These patients have an increased risk of distant metastases^3,4^. Therefore, the combination of p16 immunohistology and HPV DNA detection (or HPV RNA, which is technically more difficult) should be considered as the gold standard. Clinically, the heterogeneity of dichotomous testing for both HPV high-risk DNA and p16 should be considered, in addition to the inherent heterogeneity of HPV-viral load^5–9^.

A more complex picture emerges as recent studies indicate that HPV association in OPSCC seems to be more heterogeneous^10^. For instance, single-nucleotide polymorphisms in HPV-16 subtypes may be associated with a worse outcome in HPV-associated OPSCC^11^. Other studies focusing on the immune microenvironment showed that highly inflamed HPV-associated OPSCC cases have a favorable outcome^12,13^. In line with these findings, a retrospective analysis of tissue specimens from the TROG12.01 and de-ESCALaTE trials found that CD103 abundance identified patients with improved outcomes^14^. Previous studies have shown that within subpopulations of OPSCC, using p16 as surrogate for HPV association, information from regular H&E stains could be used to stratify OPSCC patients^15,16^.

Recently, we developed a deep learning-based approach to predict the HPV association using scans of regular H&E stains^17^. The previous approach included a smaller dataset with a focus on prediction of HPV-status and comparison to human observers. However, it lacked information on treatment modalities and different disease stages, as well as different tumor locations (primary/lymph node metastases). In this study, our objective is to develop a modular and computationally efficient algorithm designed to stratify OPSCC patients with higher accuracy compared to conventional HPV testing. To achieve this, we include multiple clinical cohorts, which encompass a wide range of disease stages, primary tumors, and metastases. Subsequently, we evaluate the algorithm’s performance against the established gold standard in various clinical scenarios.

## Results

### Patient characteristics

**Fig. 1:**
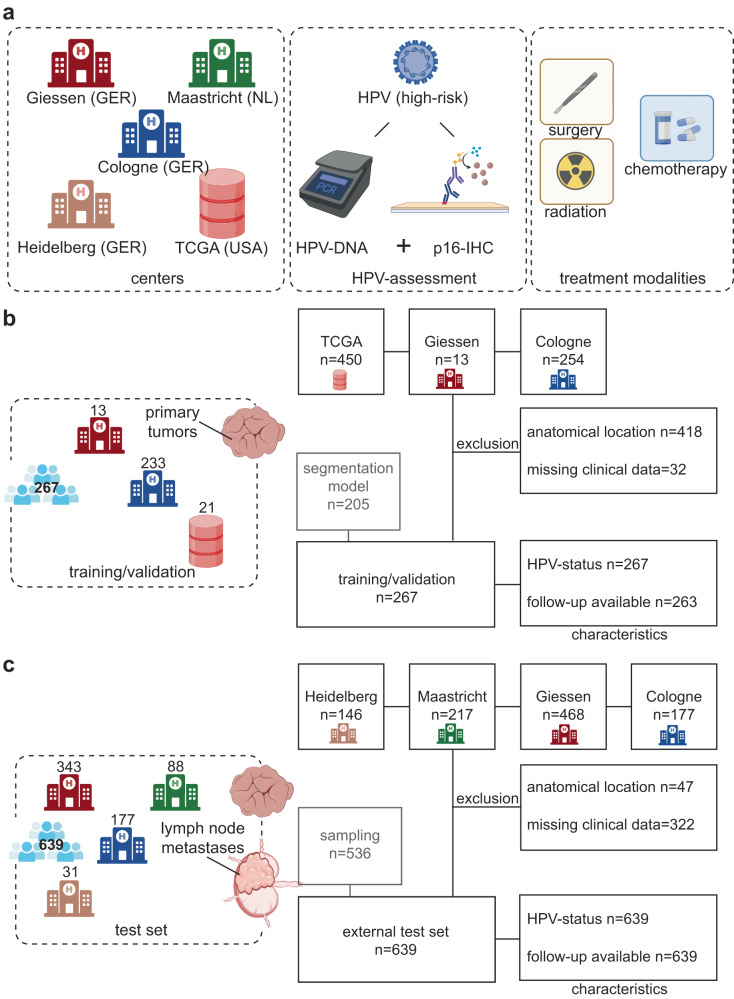
Study design and overall concept. **a** Patients from Cologne (C, GER), Giessen (G, GER), Maastricht (M, NL), Heidelberg (H, GER) and TCGA (T, USA; database) were included in the study (*n* = 906). HPV association was defined as either dichotomous HPV-DNA and p16 IHC status if both markers were available or by p16/detection of high-risk HPV-DNA. All patients received standard treatment of care. **b** CONsolidated Standards Of Reporting Trials (CONSORT)-like flow chart representing the study population of the training/validation cohort of 267 patients. Cases that could not be identified as OPSCC or with missing information on HPV-status were excluded. There were four cases where follow-up data were not available in the training cohort, which were used for training the model for HPV association, but not for survival analysis. (**c**) CONSORT-like flow chart representing the study population of the external test set of 639 patients. . Both primary tumors and lymph node metastases were included in the test set.

The study population is derived from four centers and one database (Fig. 1a, Supplementary Table 1, *n* = 906). 192 (21.2%) patients were female and 714 (78.8%) were male, the mean age was 59.4 years (interquartile range, IQR = 12.9 years). There were 477 (52.7%) patients with stage I and II disease, compared to 396 with stage III-IV disease (43.7%) and 33 (3.6%) with incomplete data on disease stage (UICC 8^th^). 364 patients (40.2%) received adjuvant radio-/chemotherapy and surgical treatment (S(C)RT), compared to 348 (38.4%) receiving definitive radio-/chemotherapy ((C)RT), as well as 129 (14.2%) patients receiving surgery alone (ST). There were 65 patients with incomplete treatment data (7.2%). All patients were analyzed for HPV-status, using either p16, HPV high-risk DNA or both (referred to as dichotomous p16/HPV-DNA testing). In total, there were 798 patients where dichotomous HPV-status was available (88.1%). Overall, there were 378 HPV-positive cases (41.7%), and 528 HPV-negative cases (58.3%).

### Building a deep learning model to predict HPV association and evaluating the performance

In continuation of our previous methodology for predicting the association of Human Papillomavirus (HPV) using histological samples^17^, we adopted a comparable approach. Specifically, we constructed a Feature Pyramid Network (FPN) with a ResNet-18 encoder to perform semantic segmentation of viable tumor regions. Subsequently, we classified the extracted tumor tiles based on their HPV association using an additional ResNet-18 network. To be data-efficient, we used a training dataset of 267 patients from two centers and one database (Fig. 1b).

**Fig. 2:**
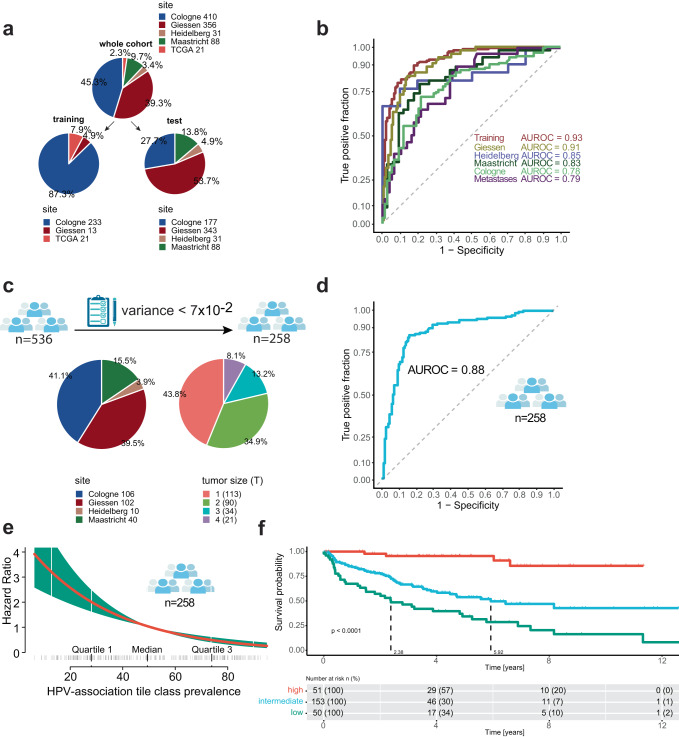
Performance of the model to predict HPV association for six different patient populations. **a** Pie charts for patient characteristics for the training cohort (left panel) and test cohort (right panel). The number besides the panel indicates the number of patients originating from each center. **b** Area under the receiver operator curve (AUROC) for six different patient populations. Cologne (*n* = 177), Giessen (*n* = 240), Maastricht (*n* = 88), Heidelberg (*n* = 31), and lymph node metastasis (*n* = 103) which originated from the Giessen site and were independent to the training cohort. **c** Overview of the study population after applying a threshold of cases with a variance below $7x{10}^{-2}$. **d** Area under the receiver operator curve (AUROC) for cases that were filtered by the threshold of variance. **e** Visualization of the Cox proportional hazards model for tile class prevalence of the HPV-positive class. The red line indicates the smoothened function, the vertical lines at the horizontal axis indicates individual patients with a given risk. The green bar indicates the error of the function. **f** Kaplan–Meier curve of *n* = 258 patients stratified for tile class prevalence of the HPV-positive class. A Cox proportional hazards model was used to compare survival curves.

The algorithm achieved an overall performance of area under the receiver operator curve (AUROC) of 0.83 (95% CI = 0.77–0.9; *n* = 639) for predicting HPV association on samples that were completely independent of the training dataset (Fig. 1c, test set). The sensitivity was 0.78 (95% CI = 0.67–0.89) and the specificity was 0.8 (95% CI = 0.62–0.99) with an accuracy of 0.77 (95% CI = 0.69–0.85). The performance on the training dataset was AUROC = 0.93 (sensitivity = 0.9, specificity = 0.83 and accuracy = 0.86; *n* = 267) (Supplementary Table 2, Fig. 2b).

Next, we evaluated whether we could increase the performance of the model to maximize its sensitivity by applying a fixed threshold on the variance of the probability of the HPV-positive class (Supplementary Figs. 2a, b; graphically explained). We therefore randomly sampled three times 30 cases out of a collection of 536 cases of primary tumors, originating from the test set (Fig. 1c; sampling), to avoid overfitting our data. These cases were filtered by a given variance of the probability of the HPV-positive class (Supplementary Fig. 2d). By filtering cases with a threshold below $7\,{\rm{x}}\,{10}^{-2}$, the best tradeoff between sensitivity (0.89 ± 0.04), specificity (0.8 ± 0.06), accuracy (0.83 ± 0.05) and the AUROC (0.86 ± 0.04) could be observed (Supplementary Table 3, Supplementary Fig. 2d). By applying this fixed threshold, we extracted a total of 258 cases (48.1%) from a collection of 536 patients (Fig. 2c). Here, an AUROC of 0.88 could be achieved (Fig. 2D; sensitivity = 0.85, specificity = 0.84 and accuracy = 0.85, *n* = 258).

### Prognostic relevance of determining HPV association using deep learning and a binary classification

We then investigated the prognostic role of predicting HPV association within this cohort and compared it to regular testing of HPV status. By setting a threshold of 50% of the tile class prevalence (Supplementary Figs. 2a, b), and mimicking the binary classification of HPV-positive/negative, a five-year overall survival rate of 80% [95% CI = 71–90%] for patients classified as HPV-positive was observed (Supplementary Fig. 3a), which was similar to the five-year overall survival rate of standard HPV testing of 80% [95% CI = 71–90%] (Supplementary Fig. 3b). Misclassification of the HPV-status was not to the disadvantage of patients when stratified for survival (Supplementary Figs. 3c, d).

### Granular patient prognostication by predicting HPV association

To resolve the prognostic value of predicting HPV association, we used a Cox proportional hazards model. Here, the tile-class prevalence as a measure of HPV association (Supplementary Figs. 2a, b) inversely correlated with survival (Likelihood-ratio test (LRT) = 49.23, *p* < 0.001, employing a chi-square distribution; concordance index (c-index) = 0.71; Fig. 2e). Having shown that the prediction of HPV association strongly correlated to survival, we applied a three-tier threshold (high, referred to highly HPV-associated; low and intermediate above/below 20% and in between). By following this, we could identify patients with a five-year survival rate of 96% [95% CI = 90-100] (Fig. 2f, high) outperforming the previously mentioned 80% five-year survival rate of the gold standard of HPV testing [95% CI = 71-90%]. For patients classified as intermediate, the five-year survival rate was 54% [95% CI = 45–65%], and for patients classified as low 34% [95% CI = 23–52%]. A multivariate analysis including several clinical variables (therapy, tumor size, nodal status, smoking history, and sex) confirmed the prognostic superiority of predicting HPV association using OPSCCnet (high: HR = 0.15 [95% CI = 0.05–0.44], *p* < 0.001, Cox proportional hazards model; intermediate: HR = 0.43 [95% CI = 0.26–0.7], *p* = 0.043, Cox proportional hazards model; low: reference; *n* = 211; Supplementary Fig. 4a).

The gold standard of HPV testing showed less prognostic relevance of HPV testing when compared to the algorithm (LRT = 39.72, *p* < 0.0001, employing a chi-square distribution; c-index = 0.65), which was underlined by the results of a multivariate analysis (HPV testing: HPV-positive HR = 0.29 [95% CI = 0.15–0.54], *p* < 0.001, Cox proportional hazards model; *n* = 211; Supplementary Fig. 4b).

### Developing a combined scoring of the variance of the tile class probability and the tile class prevalence for patient prognostication

By using the variance of the tile class probability (Supplementary Fig. 2a, b) we could increase the sensitivity and specificity of determining HPV association in about 50% of the study population in the external test set (*n* = 258), outperforming regular HPV-status in selecting patients with an improved prognosis. We therefore reasoned to investigate the prognostic relevance of combing variance of the tile class probability and the tile class prevalence together (Supplementary Figs. 2a, b), leading to a combined HPV association score (referred to as combined score; Supplementary Fig. 2c**;** Eq. (3)).

**Fig. 3:**
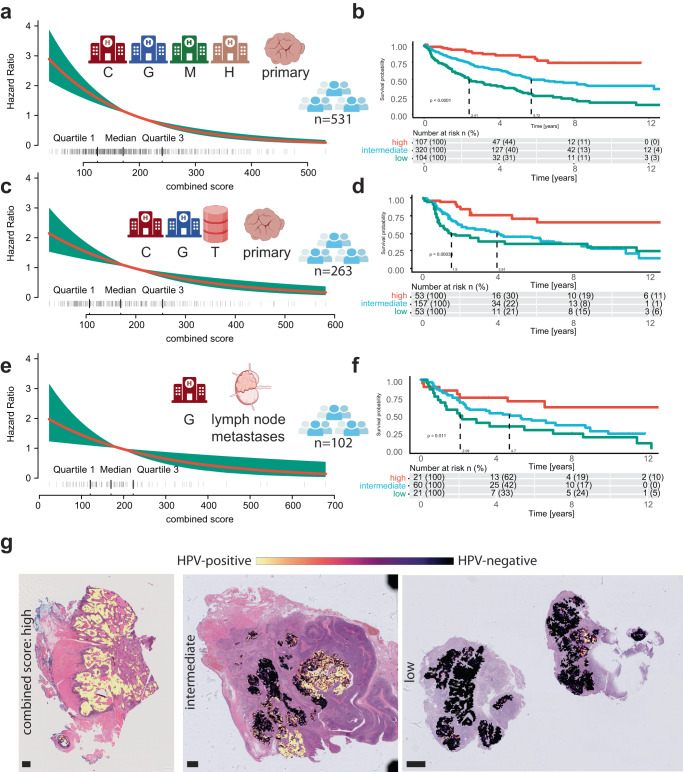
Prognostic relevance of predicted HPV association. **a** Hazard ratio plot of the predicted HPV association and overall survival for patients from the test cohort: Cologne (C; *n* = 177), Giessen (G; *n* = 240), Maastricht (M; *n* = 88) and Heidelberg (H; *n* = 31). **b** Corresponding Kaplan–Meier curve for the same population as depicted in A (*n* = 531). The cohort is divided into three separate groups by the combined score. A Cox proportional hazards model was used to compare survival curves. **c** Hazard ratio plot of the predicted HPV association and overall survival for patients from the training cohort: Cologne (C; *n* = 233), Giessen (G; *n* = 13), and TCGA (T; *n* = 21), with a total of *n* = 263 patients. **d** Corresponding Kaplan Meier curve for the same population as depicted in C (*n* = 263). The cohort is divided into three separate groups by the combined score. A Cox proportional hazards model was used to compare survival curves. **e** Hazard ratio plot of the predicted HPV association and overall survival for patients from the test cohort, exclusively containing lymph node metastatic samples: Giessen (G; *n* = 102). **f** Corresponding Kaplan–Meier curve for the same population as depicted in E (*n* = 102). The cohort is divided into three separate groups by the combined score. A Cox proportional hazards model was used to compare survival curves. **g** Histological image of three cases with corresponding classes of the combined score. The scale bars have a length of 0.052 mm.

For the external test set, filtered for primary tumors, there was a strong association between the combined score and overall survival, by again using a Cox proportional hazards model (LRT = 62.26, *p* < 0.001, employing a chi-square distribution, *n* = 531; Fig. 3a). We then divided the patients into three separate groups (high HR = 0.17 [95% CI = 0.10–0.29]; intermediate HR = 0.49 [95% CI = 0.37–0.66]; low: reference) with distinct five-year survival rates (high: 85% [95% CI = 77–93%], intermediate: 56% [95% CI = 50–63%], low: 34% [95% CI = 25–45%]; Fig. 3b). A similar association, given the overall lower five-year overall survival rate of the training cohort, was observed in the training cohort (LRT = 24.86, *p* < 0.001, employing a chi-square distribution, *n* = 263; Fig. 3c). The five-year survival rates were 71% [95% CI = 56–90%] for the high group, 45% [95% CI = 36–56%] for the intermediate group and 31% [95% CI = 20–49] for the low group (high: HR = 0.24 [95% CI = 0.12–0.48]; intermediate HR = 0.68 [95% CI = 0.45–1]; low: reference; Fig. 3d).

In addition, the generalizability of the prognostic relevance was confirmed by analyzing tissue of lymph node metastases, although the intermediate group did not reach a level of significance (LRT: 13.39, *p* = 0.004, employing a chi-square distribution, *n* = 102, Fig. 3e) high HR = 0.28 [95% CI = 0.12–0.67]; intermediate HR = 0.65 [95% CI = 0.37–1.14], *p* = 0.12, employing a chi-square distribution; low: reference (Fig. 3f).

### Prognostication of patient subpopulations using the combined score of HPV association

**Fig. 4:**
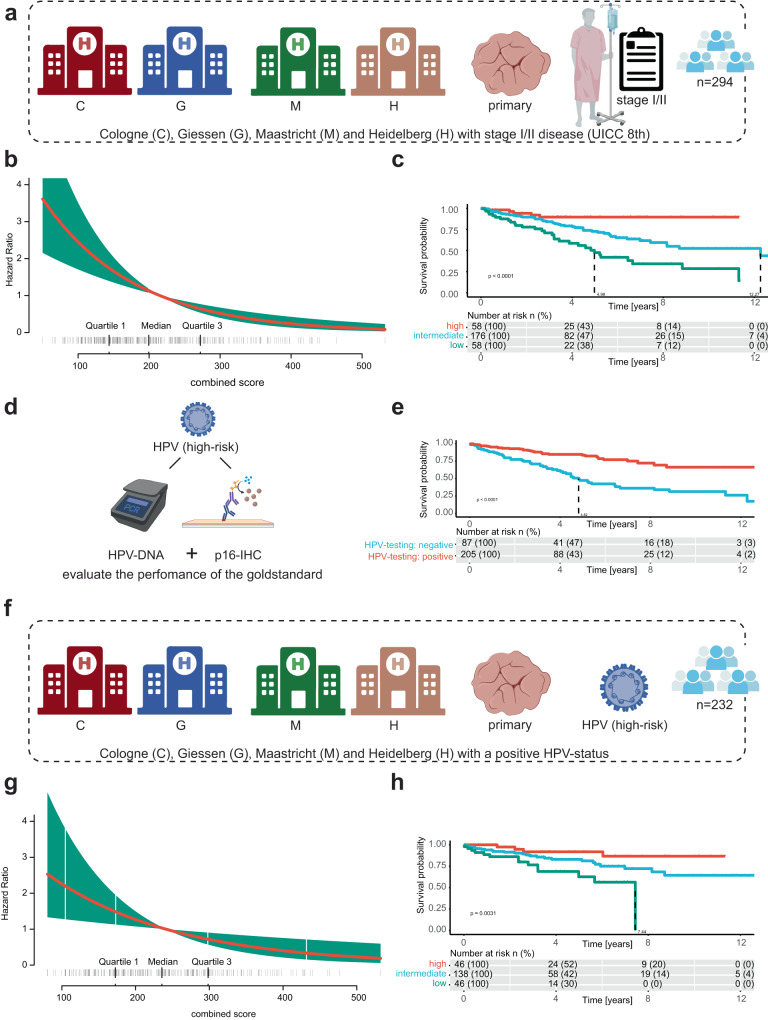
Prognostic significance of the predicted HPV association for stage I/II patients compared to gold-standard. **a** Patients with early-stage disease I/II (Union for International Cancer Control; UICC 8th) from different centers of the test cohort are included for the subsequent analysis. **b** Hazard ratio plot of the selected patients with stage I/II disease. **c** Kaplan–Meier curve for the survival benefit of stage I/II patients according to the combined score. A Cox proportional hazards model was used to compare survival curves. **d** Schematic, illustrating that for comparison HPV testing, with both HPV-DNA assessment and p16 status (dichotomous testing) was used. **e** Kaplan–Meier curve for regular HPV testing. A Cox proportional hazards model was used to compare survival curves. **f** Schematic illustration of the filtering criteria for the subsequent analysis. Cases from Cologne, Giessen, Maastricht, and Heidelberg that were tested as HPV-positive (*n* = 232). **g** Hazard ratio plot of the selected patients with stage a positive HPV-status (*n* = 232). **h** Kaplan–Meier curve for the combined score of patients tested as HPV-positive (*n* = 232). A Cox proportional hazards model was used to compare survival curves.

Having shown that predicting HPV association allowed accurate stratification of OPSCC patients, we next evaluated the prognostic relevance of the combined score in a subgroup of patients with early-stage disease (stage I/II, UICC 8th, *n* = 294; Fig. 4a). Particularly this group of patients may qualify for potential treatment de-escalation strategies. Again, there was a strong inverse correlation of the combined score and survival (LRT = 29.3, *p* < 0.001, employing a chi-square distribution, *n* = 294; Fig. 4b).

Patients with a high score had a HR = 0.15 [95% CI = 0.06–0.38], *p* < 0.001, derived by a Cox proportional hazards model, compared to patients in the intermediate group with a HR of 0.46 [95% CI = 0.29–0.73], *p* < 0.001, Cox proportional hazards model (Fig. 4c). The five-year survival rates were 90% [95% CI = 82–99%] for patients classified as high, 73% [95% CI = 65–82%] for patients classified as intermediate and 48% [95% CI = 35–65%] classified as low. A multivariate analysis showed that the combined score was an independent predictor among several variables (high, HR = 0.22 [95% CI = 0.03–0.59], *p* = 0.003, Cox proportional hazards model; intermediate, HR = 0.53 [95% CI = 0.30–95], *p* = 0.033, Cox proportional hazards model, *n* = 253; Supplementary Fig. 5a). Within the same patient population, the performance of the gold standard of HPV-DNA/p16 combination (Fig. 4d) achieved a HR = 0.31 [95% CI = 0.2–0.48], Fig. 4e, with a five-year survival rate of 83% [95% CI = 78–90] for HPV-positive cases and 48% [95% CI = 37–61] for HPV-negative cases. The LRT was 28.1, *p* < 0.001, employing a chi-square distribution, and a multivariate analysis revealed a HR of 0.26 for HPV-positive cases [95% CI = 0.15–0.45], *p* < 0.001, Cox proportional hazards model, *n* = 253 (Supplementary Fig. 5b).

Within the training cohort, adjusted for cases with stage I/II disease (*n* = 151), the combined score could stratify patients with a HR = 0.13 [95% CI = 0.04–0.5], *p* = 0.002, (Cox proportional hazards model) in the high group and HR = 0.54 for intermediate group [95% CI = 0.29–1.1]; *p* = 0.072, Cox proportional hazards model (Supplementary Fig. 6a). The five-year survival rates were 78% [95% CI = 58–100%] for patients classified as high, and 65% [95% CI = 53–79%] for patients classified as intermediate. For the low group, the five-year survival rate was 48% [95% CI = 31–75%]. A multivariate analysis yielded a HR of 0.10 [95% CI = 0.02–0.50], *p* = 0.005, derived by a Cox proportional hazards model for the high group and HR = 0.43 [95% CI = 0.16–1.14], *p* = 0.091, derived by a Cox proportional hazards model for the intermediate group (Supplementary Fig. 6b; *n* = 136). The HR of HPV testing was 0.38 [95% CI = 0.15–0.95], *p* < 0.001, derived by a Cox proportional hazards model for HPV-positive cases Supplementary Fig. 6c. A multivariate analysis of regular HPV testing showed a HR for HPV-positive cases of 0.22 [95% CI = 0.09–0.53], *p* < 0.001, Cox proportional hazards model, *n* = 136 (Supplementary Fig. 6d).

To draw a more complete picture of the performance to stratify patients using the combined score, we next selected patients of the test set for advanced stage disease (stage III/IV, UICC8^th^; Supplementary Fig. 7a; combined score, LRT = 9.5, *p* = 0.002, employing a chi-square distribution, *n* = 219, c-index = 0.57; Supplementary Fig. 7b). Patients with a high combined score had a HR of 0.48 [95% CI = 0.27–0.86], *p* = 0.013, *n* = 219 (Supplementary Fig. 7c), while a level of significance could not be reached for the intermediate group (HR = 0.81, [95% CI = 0.54–1.21], *p* = 0.3). For regular HPV testing a level of significance could not be reached (HPV-positive, LRT = 2.77, *p* = 0.1, employing a chi-square distribution, c-index = 0.51, HR = 0.53 [95% CI = 0.23–1.2], *p* = 0.12, Cox proportional hazards model; Supplementary Fig. 7d). A multivariate analysis showed a significant prognostic association of the high combined score, HR = 0.42 [95% CI = 0.22–0.77], *p* = 0.006, derived by a Cox proportional hazards model and no significant association for the intermediate group HR = 0.81 [95% CI = 0.52–1.26], *p* = 0.35, Cox proportional hazards model (Supplementary Fig. 7e; *n* = 195).

In addition, we selected only HPV-positive cases (Fig. 4f) and applied the combined score to stratify these patients. Here, the combined score showed an LRT = 9.3 (*p* = 0.002, employing a chi-square distribution, *n* = 230, c-index = 0.63; Fig. 4g). By again dividing patients using the same three-tier threshold, patients with a high score had a HR = 0.18 [95% CI = 0.06–0.56], *p* = 0.003, derived by a Cox proportional hazards model and patients with an intermediate score HR = 0.46 [95% CI = 0.24–0.90], *p* = 0.022, Cox proportional hazards model (Fig. 4h). This resulted in a five-year overall survival rate of 92% [95% CI = 84–100%] for patients within the high group, 83% [95% CI = 76–90%] for patients in the intermediate group and 63% [95% CI = 47–84%] for the low group. Consistently, by following a multivariate approach, the analysis showed a significant prognostic association of the high combined score with HR = 0.21 [95% CI = 0.05–0.87], *p* = 0.032, derived by a Cox proportional hazards model, and no significant association for the intermediate group HR = 0.54 [95% CI = 0.24–1.24], *p* = 0.128, Cox proportional hazards model (Supplementary Fig. 7e; *n* = 196).

To this end, we explored visual correlates of misclassified cases and provided their clinical characteristics. We chose four cases, namely with the highest tile-class prevalence, but which were falsely classified as HPV-positive (false-positive) or that were falsely classified as HPV-negative (false-negative). Interestingly, two cases being falsely classified as HPV-positive were found to be p16 positive but HPV-DNA negative (Supplementary Fig. 9a) with an overall survival of 6.6 and 12.3 years, respectively. Two cases that had been falsely classified as HPV-negative were both found to be HPV-DNA/p16 positive but had an overall survival of 0.16 years and 0.42 years, respectively (Supplementary Fig. 9b).

## Discussion

Here, we present an approach of segmenting viable tumor areas following a subsequent classification of tumor patches. The main advantage of this approach is that it allows visual inspection of tissue areas that are subsequently used for classification. As a result, we have better control over the input data used in the subsequent classification of the Human Papillomavirus (HPV) association. To demonstrate the generalizability of our approach, we include lymph node metastases and primary tumors from different centers and treatment modalities in our external test set. The strong association of predicting HPV association and prognosis is confirmed in early- and late-stage disease, and it could also be used to stratify patients who are annotated as HPV-positive – identified by regular HPV testing.

Throughout this study, we are consequently using the same threshold for each cohort (high, referred to highly HPV-associated; low and intermediate above/below 20% and in between). Moreover, we provide evidence that both the tile class prevalence and the combined score are strongly correlating (inversely) to prognosis – irrespective of a specific threshold. However, by using this three-tier threshold, we aim to make the results comparable, allowing subsequent multivariate analysis. In this context, it is noteworthy to acknowledge that when analyzing patients with advanced stage disease, the intermediate group does not exhibit a statistically significant different outcome. However, unlike the conventional testing for HPV, which does not show a significant prognostic value within this subgroup of advanced stage disease, the group identified as having a high combined score demonstrates a statistically significant improvement in prognosis. From a clinical standpoint, patients with advanced stage disease would not typically be considered for treatment de-escalation, and it appears that conventional HPV testing also fails to differentiate these patients for prognostication. Therefore, we propose that by employing OPSCCnet, a certain percentage of patients could be identified with a slightly decreased or beneficial prognosis, which could subsequently be utilized for ongoing clinical monitoring of these individuals. Importantly, we believe that by utilizing a three-tier classification model, as opposed to the conventional binary HPV-testing, this would be considered a more detailed biomarker that enhances the resolution of patients’ prognosis, enabling the consideration of potential treatment de-escalation as well as surveillance.

Having built an algorithm to predict HPV association using 267 cases (training/validation dataset) that are predominantly determined by both p16 and HPV-DNA (246 cases / 92.1% where dichotomous HPV-status is available for 21 cases / 7.9% from the TCGA database), we believe that this ground truth is most versatile, given the discrepancies of p16/HPV-DNA testing and the associated distinct prognostic association with discordant p16/HPV-DNA status^3,18–20^. By employing a random subsampling approach to determine the variance of the tile class probability in our dataset and to improve its test-metrics, we sought to address generalizability and robustness. Random subsampling helps in obtaining an estimate of the threshold that is not overly influenced by specific characteristics of the entire cohort. Using the entire dataset to define the threshold could potentially lead to overfitting, where the threshold is tailored too closely to the peculiarities of the dataset itself and may not accurately generalize to other similar datasets or populations. By randomly subsampling the data, we aim to create multiple representative subsets that capture the inherent variability within the dataset. Our approach of filtering cases with a certain threshold of the variance of the tile class probability would limit the application of declaring HPV association accurately to about 50% of patients. In comparison, other techniques, such as molecular assessment of oncogenic alterations require a level of tissue quality and recent efforts have made the necessity of quality control in computational pathology evident^21^. Arguably, we incorporate a threshold reflecting a range where the predictions of the algorithm are most accurate. At the same time, recent studies by Lang Kuhs et. al.^11^ underline the heterogeneity of HPV-positive OPSCC, which is also in line with previous findings showing that HPV viral load is association with prognosis in OPSCC^5^. We therefore argue that our approach of using the tile class prevalence, as well as the variance of the class probability (combined score; Eq. (3)) as prognostic marker has a biological correlate of HPV heterogeneity. A very recent study by Sid. et. al. defines a subpopulation of OPSCC HPV positive tumors where malignant cells retained viral gene expression (“HPVon”), as well as cells that phenotypically suppress viral gene expression (“HPVoff”)^22^. Although we currently have no evidence whether these results correlate to the results of our analysis, future studies may analyze these patterns and compare whether the results of OPSCCnet might recapture this biological phenomenon.

However, it should be considered that once larger, well annotated datasets of OPSCC emerge, this would warrant further investigation of declaring HPV subgroups based on HPV genotypes^11^. Potentially, these may then be further supplemented with other deep learning techniques, including multiple-instance-learning – a method of weakly supervised learning^23,24^. Currently, the presented method of using a supervised learning approach and filtering criteria appear versatile in identifying patients with improved prognosis. Notably, a simple comparison of the performance of declaring HPV association might not be the ultimate goal, given the heterogeneity of HPV association shown by recent studies^10,22^. Instead, machine learning applications should be deployed to identify OPSCC patients with a distinct clinical course accurately. Surprisingly, we found that two cases initially misclassified as HPV positive by the OPSCCnet were p16 positive and HPV-DNA negative (classified as HPV negative). This indicates a false-positive result from applying the OPSCCnet algorithm. Additionally, two cases that were determined to be HPV positive (p16 positive and HPV-DNA positive) were classified as HPV negative by OPSCCnet. These cases had a significantly shorter overall survival of 0.16 and 0.42 years compared to the overall prognosis of HPV-positive patients. It is important to note that all these tumors were located at the tonsils. This highlights the need to accurately distinguish HPV status and stratify patients when evaluating algorithm performance. Therefore, we believe that by introducing our combined score, we can more accurately identify HPV-associated OPC and improve prognosis compared to standard HPV testing methods.

Nevertheless, it should be considered that our study is not without limitations. The first limitation is that the data were primarily collected from European centers and patient characteristics may vary from other geographical regions. This may indeed apply to any predictive biomarker and hence needs to be investigated in additional cohorts of various ethnicities and geographical regions. Another limitation is that we collected the samples retrospectively. This results in confounders which would need to be validated by a randomized trial – including de-escalation strategies for patients stratified using HPV prediction.

Given the strong prognostic association of predicting HPV association and overall survival, future studies exploring treatment de-escalations or prognostic biomarkers within OPSCC can make use of the freely available algorithm, which we release as “OPSCCnet”, a combination of tumor detection and tumor classification of HPV association. As there is an ongoing effort to explore the exact value of p16 as single biomarker of HPV association, in comparison to both p16 and HPV-DNA status (dichotomous HPV testing) to declare HPV association, these studies may benefit from incorporating results from the OPSCCnet.

In conclusion, we develop and validate a fully automated modular algorithm – referred to as OPSCCnet – which allows patient stratification using regular H&E virtual-whole-slide images. The algorithm can be run using regular clients outperforming the gold standard of p16/HPV-DNA testing. Together, this tool may allow the community to stratify OPSCC patients at a highly granular level.

## Methods

### Study design and patients

The study design is shown in Fig. 1. 906 patients with OPSCCs from four centers and one database were enrolled in this retrospective study. 263 patients were used as training cohort (Fig. 2A; Supplementary Table 1). Patients from Cologne, Giessen, Heidelberg, and Maastricht were diagnosed with primary squamous cell carcinoma of the oropharynx (ICD code C10, International Classification of Diseases for Oncology) and treated at the given local center between 2005 and 2019. The study was conducted in accordance with the Declaration of Helsinki, and the protocol was approved by the regional ethics committees (Giessen: AZ 95/15, dated October 19, 2015; Cologne: AZ 19–1288_1, dated February 3, 2020). Informed written consent was obtained from each subject. Patient characteristics were recorded prospectively by the Giessen cancer registry database (GTDS), as well as from the cancer registry database of the Center for Integrated Oncology (CIO), Cologne. H&E stained tumor samples were provided by the Tissue Bank of the National Center for Tumor Diseases (NCT) Heidelberg, Germany in accordance with the regulations of the tissue bank and the approval of the ethics committee of Heidelberg University (S-207/2005 and S-786/2021)^25^. Ethical approval for use of the Maastricht samples was granted by the local ethical committee under the study number METC-2021 2658. All patients were treated in accordance with approved guidelines by either surgery alone (ST) or upfront surgery and concomitant (chemo)radiotherapy (S(C)RT) or definitive chemoradiotherapy ((C)RT). Overall survival was defined as the time after initial diagnosis to death from any cause.

### HPV-status

HPV-status was determined by presence of HPV high-risk DNA, p16 or combination of both (dichotomous HPV-status), if both markers were available. Corresponding p16 status on tumor tissue was assessed by staining and scoring for p16, according to EORTC guidelines, based on cytoplasmatic and nucleic p16 expression in 70% of tumor cells^26,27^. Isolation of tumor DNA and HPV genotyping was performed as described previously^28^.

### Evaluating the best model for accurate prediction of HPV association

To choose the best model, we tested five different network architectures. First, we build a training dataset (500 cases) and a test dataset (216 cases) from a total of 716 patients in total (Supplementary Fig. 1A). Then, we evaluated the performance of ResNet-50, ResNet-18, densenet-201, mobilenetv2, inceptionv3 on these samples (Supplementary Fig. 1B). All networks were pretrained models trained on ImageNet^29^. Together, there were 1.000.000 224 × 224 image tiles within this training set.

### Building and applying a deep learning-based HPV prediction-algorithm

#### Semantic segmentation

We built a Feature Pyramid Network (FPN) with a ResNet-18 encoder for semantic segmentation of viable tumor areas using segmentation models (PyTorch, https://github.com/qubvel/segmentation_models.pytorch, author: Pavel Yakubovskiy). Image tiles of 1024 × 1024 with a pixel size of 2.2 μm/pixel were extracted, exclusively from tissue carrying parts of the whole-slide-image (WSI), which were classified using a simple tissue classification network built within QuPath^30^. We trained the FPN model using 3924 image tiles originating from 205 cases from the training dataset (Fig. 1b; segmentation). The performance metrics of the segmentation were intersection over union (iou) iou = 0.94 / 0.9 for the test dataset (dataset / image based) and iou = 0.87 / 0.86 for a holdout test set (dataset / image based). Training was performed with albumentations for augmentation and in particular staintools for color augmentation using Reinhard, Vahadane und Macenko stain normalization methods^31–33^.

### Image classification, hyperparameters

The ResNet-18 model for classification was trained using a dataset with a class prevalence of 1.03 for HPV-positive and 0.97 for HPV-negative, given that we built a balanced dataset by randomly selecting image tiles of the 267 cases of the training/validation data to a total of 1,000,000 images (224 × 224 pixels). The batch size was 32 and the dataset was split into training and validation sets (80/20), resulting in 800,000 files for training and 200,000 files for validation. Data augmentation techniques such as random horizontal and vertical flipping and rotation were applied to the training data. Moreover, we used a dedicated color augmentation strategy by applying H&E stain normalization. We applied a non-linear activation function (sigmoid activation function) to the output of the final dense layer of the ResNet-18 given the binary nature of the classification task. Adam was used to update the model’s parameters as optimizer and a cross entropy was used as loss function. The learning rate was scheduled to decrease over time using an initial learning rate of 0.01, decay steps of 10,000 and a decay rate of 0.9. The model’s performance on the validation set was monitored using accuracy and the training process was stopped early if the validation accuracy would not improve for eight consecutive epochs. The total number of training steps in each epoch was 25000.

### Screening for the best suited deep learning network architecture

To choose an architecture of a deep-convolutional neural network that would yield the best results, we randomly selected 500 cases from the whole cohort and evaluated the performance on 216 independent cases (Supplementary Fig. 1a). For the screening of the most appropriate architecture, we have optimized the hyperparameters for ResNet-50 using a smaller fraction of the total cases. ResNet-18 archieved best results (AUROC 0.84; Supplementary Fig. 1b). The ResNet-18 model that was trained for screening purpose was exclusively used in the screening setting and not applied during the study – where we trained an independent network using a training dataset with fewer cases in total, but were we extracted a higher number of image tiles per case. Generally, ResNet-18 is an architecture that provides a cost-effective computational solution while exhibiting a high degree of generalizability to unseen datasets, archiving good performance metrics when trained and applied on biomedical data^34,35^.

### Classification of tumor tiles

For classification of tumor tiles, patches with a centroid within tumor areas were extracted (224 × 224 pixels), followed by stain normalization^31^. For training, but not for inference, overlapping tiles were extracted. The resulting image tiles were then used to train a ResNet-18 using Tensorflow. We chose ResNet-18 model that was pre-trained on the ImageNet dataset. The top layers of the ResNet18 model, originally designed for multi-class classification, were excluded to enable the integration of customized layers. A global average pooling layer was added to the output of the base model. Subsequently, a dense layer with a sigmoid activation function was appended to the global average pooling layer to generate binary class probabilities. For training, all layers have been optimized. All code, including requirements and detailed information on the software is available at https://github.com/OPSCCnet/OPSCCnet.

### Whole-slide-image scanning, computing structure

Slides from Cologne, Giessen, Heidelberg and Maastricht were scanned using a NanoZoomer S360 Digital slide scanner with a pixel size of 0.23 μm/pixel (*n* = 884). Cases from the TCGA database were scanned at 20X magnification using Aperio slidescanners (*n* = 22; https://portal.gdc.cancer.gov/).

### Combined score and variance of the tile class probability

The tile class prevalence (TP) and the variance of the probability of the HPV-positive class (*s*^2^) are explained schematically in Supplementary Figs. 2a, b. Given the increasing evidence of heterogeneity within HPV-associated OPSCC tumors, we calculated a combined score (Eq. (3)), which is explained schematically in Supplementary Fig. 2c.

Variance; Eq. (1):

1 $${s}^{2}=\frac{{{\sum }_{i=1}^{n}\left({x}_{i}-\bar{X}\right)}^{2}}{n-1}$$

Tile class prevalence (TP) for HPV-positive (Hpos); Eq. (2):

2 $${TP}=\left(\frac{{\rm{Hpos}}\left(n\right)}{n}\right)* 100$$

Combined score; Eq. (3):

3 $${combined}\,{score}={TP}* \log 2\left(\frac{1}{{s}^{2}}\right)$$

### Performance metrics and threshold

To evaluate the performance of the algorithm to determine HPV-status, we used either dichotomous HPV-status (p16/HPV-DNA) - where available - or either p16 or HPV-high risk DNA as ground truth. We have calculated the Youden index for the threshold of the classification of HPV-positive cases using the subset of validation cases as part of the training dataset to avoid overfitting our data. This has been calculated separately for the screening of architectures (methods) and the final ResNet-18 model. For the three-tier threshold of the combined score we applied the same criteria (high, referred to highly HPV associated; low and intermediate above/below 20% and in between).

### Statistical analysis and computation

Univariate and multivariate analysis were conducted using a Cox proportional hazards model. Likelihood-ratio tests were performed as part of a Cox proportional hazards model (also referred to as Cox-regression). A *p*-value of less than 0.05 was considered statistically significant. All tests were two-sided. Statistical analysis was done with R and Python. All computation was performed using dual NVIDIA RTX 3090 GPUs.

### Reporting summary

Further information on research design is available in the Nature Research Reporting Summary linked to this article.

### Supplementary information

Supplementary information

Reporting Summary

## Data Availability

The datasets from the four centers cannot be made publicly available because of privacy requirements of the participating medical centers. All code for inference, including weights and software specifications/installation guides, is available online (https://github.com/OPSCCnet/OPSCCnet). Users can download OPSCCnet and run the analysis using their own data.
