# Supplementary material for: Predicting HPV association using deep learning and regular H&E stains allows granular stratification of oropharyngeal cancer patients
Source: NPJ Digit Med. 2023 Aug 19;6:152. doi: 10.1038/s41746-023-00901-z (PMC10439941; doi:10.1038/s41746-023-00901-z)
Supplement: Supplementary file 1 — Supplementary information [file 41746_2023_901_MOESM1_ESM.pdf]

Supplemental Tables (1-3) and Supplemental Figures (1-8)

Supplementary Table 1		n=410	n=356	n=31	n=88	n=21	
label	**levels**	**Cologne (%)**	**Giessen (%)**	**Heidelberg (%)**	**Maastricht (%)**	**TCGA (%)**	**p**
tumor location	**Other**	241 (58.8)	211 (59.3)	0 (0.0)	26 (29.5)	14 (66.7)	<0.001
	**Tonsillar region**	169 (41.2)	145 (40.7)	0 (0.0)	62 (70.5)	5 (23.8)	
	**Oropharynx NOS**	0 (0.0)	0 (0.0)	31 (100.0)	0 (0.0)	2 (9.5)	
	**(Missing)**	0 (0.0)	0 (0.0)	0 (0.0)	0 (0.0)	0 (0.0)	
age	**Mean (SD)**	61.6 (10.1)	56.8 (16.9)	57.9 (7.7)	61.4 (8.8)	55.8 (11.8)	<0.001
	**(Missing)**	0 (0.0)	0 (0.0)	0 (0.0)	0 (0.0)	0 (0.0)	
sex	**female**	87 (21.2)	72 (20.2)	6 (19.4)	22 (25.0)	5 (23.8)	0.892
	**male**	323 (78.8)	284 (79.8)	25 (80.6)	66 (75.0)	16 (76.2)	
	**(Missing)**	0 (0.0)	0 (0.0)	0 (0.0)	0 (0.0)	0 (0.0)	
therapy	**(C)RT**	171 (41.7)	96 (27)	0 (0.0)	81 (92.0)	0 (0.0)	<0.001
	**S(C)RT**	161 (39.4)	170 (47.8)	26 (83.9)	0 (0.0)	0 (0.0)	
	**ST**	76 (18.4)	48 (13.5)	5 (16.1)	0 (0.0)	0 (0.0)	
	**(Missing)**	2 (0.5)	42 (11.7)	0 (0.0)	0 (0.0)	21 (100)	
Tumor location	**MT (metastases)**	1 (0.2)	106 (29.8)	0 (0.0)	0 (0.0)	0 (0.0)	<0.001
	**PT (primary)**	409 (99.8)	250 (70.2)	31 (100.0)	88 (100.0)	21 (100.0)	
	**(Missing)**	0 (0.0)	0 (0.0)	0 (0.0)	0 (0.0)	0 (0.0)	
tumor size (T)	**1**	258 (62.9)	171 (48.0)	4 (12.9)	12 (13.6)	3 (14.3)	<0.001
	**2**	102 (24.9)	117 (32.9)	19 (61.3)	41 (46.6)	7 (33.3)	
	**3**	33 (8.0)	50 (14.0)	7 (22.6)	11 (12.5)	3 (14.3)	
	**4**	17 (4.1)	18 (5.1)	1 (3.2)	24 (27.3)	5 (23.8)	
	**(Missing)**	0 (0.0)	0 (0.0)	0 (0.0)	0 (0.0)	3 (14.3)	
Nodal stage (N)	**0**	149 (36.3)	76 (21.3)	8 (25.8)	19 (21.6)	5 (23.8)	<0.001
	**1**	56 (13.7)	54 (15.2)	12 (38.7)	12 (13.6)	2 (9.5)	
	**2**	175 (42.7)	209 (58.7)	11 (35.5)	57 (64.8)	9 (42.9)	
	**3**	17 (4.1)	13 (3.7)	0 (0.0)	0 (0.0)	1 (4.8)	
	**(Missing)**	13 (3.2)	4 (1.1)	0 (0.0)	0 (0.0)	4 (19.0)	
grading (G)	**1**	2 (0.5)	2 (0.6)	0 (0.0)	2 (2.3)	0 (0.0)	0.061
	**2**	198 (48.3)	158 (44.4)	0 (0.0)	36 (40.9)	0 (0.0)	
	**3**	104 (25.4)	120 (33.7)	0 (0.0)	29 (33.0)	0 (0.0)	
	**(Missing)**	106 (25.9)	76 (21.3)	31 (100.0)	21 (23.9)	21 (100.0)	
smoking history	**current or former smoker**	260 (70.1)	295 (90.2)	21 (67.7)	69 (78.4)		<0.001
	**non-smoker**	111 (29.9)	32 (9.8)	10 (32.3)	19 (21.6)		
	**(Missing)**	0 (0.0)	1 (0.3)	0 (0.0)	0 (0.0)	0 (0.0)	
HPV dichotomous	**0**	200 (48.8)	266 (74.7)	0 (0.0)	4 (4.5)	0 (0.0)	<0.001
	**1**	210 (51.2)	89 (25.0)	0 (0.0)	29 (33.0)	0 (0.0)	
	**(Missing)**	0 (0.0)	1 (0.3)	31 (100.0)	55 (62.5)	21 (100.0)	
p16	**0**	174 (42.4)	243 (68.3)	0 (0.0)	34 (38.6)	0 (0.0)	<0.001
	**1**	236 (57.6)	112 (31.5)	0 (0.0)	54 (61.4)	0 (0.0)	
	**(Missing)**	0 (0.0)	1 (0.3)	31 (100.0)	0 (0.0)	21 (100.0)	
HPV-DNA	**0**	192 (46.8)	247 (69.4)	10 (32.3)	3 (3.4)	13 (61.9)	<0.001
	**1**	218 (53.2)	108 (30.3)	21 (67.7)	29 (33.0)	8 (38.1)	
	**(Missing)**	0 (0.0)	1 (0.3)	0 (0.0)	56 (63.6)	0 (0.0)	

Supplementary Table 1. Study cohort description. P-values are derived from a Chi-square test.

Supplementary Table 2

Site	n	HPV-positive (n)	HPV-negative (n)	accuracy	sensitivity	specificity	true positive	false negative	false positive	true negative	AUROC
Heidelberg	31	21	10	0.77	0.67	1.00	14	7	0	10	0.85
Maastricht	88	51	37	0.80	0.80	0.79	43	11	7	27	0.83
Cologne	177	103	74	0.74	0.72	0.78	75	29	16	57	0.78
Giessen	241	57	184	0.85	0.84	0.86	48	9	26	158	0.91
Metastases	102	28	74	0.67	0.89	0.58	25	3	31	43	0.79
training	267	118	149	0.86	0.90	0.83	106	12	26	123	0.93

Supplementary Table 2. Performance in prediction of HPV association for the different cohorts.

Supplementary Table 3

threshold	metric	first run	second run	third run	average	standard deviation
0.1	AUROC	0.82	0.88	0.79	0.83	0.04
0.09	AUROC	0.81	0.70	0.78	0.76	0.05
0.08	AUROC	0.86	0.75	0.75	0.78	0.05
**0.07**	AUROC	**0.81**	**0.85**	**0.92**	**0.86**	**0.04**
0.06	AUROC	0.84	0.83	0.93	0.87	0.05
0.1	sensitivity	0.81	0.79	0.79	0.79	0.01
0.09	sensitivity	0.73	0.79	0.67	0.73	0.05
0.08	sensitivity	0.65	0.80	0.80	0.75	0.07
**0.07**	**sensitivity**	**0.83**	**0.90**	**0.93**	**0.89**	**0.04**
0.06	sensitivity	0.80	0.69	0.90	0.80	0.08
0.1	specificity	0.79	1.00	0.75	0.85	0.11
0.09	specificity	0.93	0.69	0.80	0.81	0.10
0.08	specificity	0.92	0.73	0.80	0.82	0.08
**0.07**	**specificity**	**0.72**	**0.80**	**0.87**	**0.80**	**0.06**
0.06	specificity	0.80	0.94	0.90	0.88	0.06
0.1	accuracy	0.80	0.90	0.77	0.82	0.06
0.09	accuracy	0.83	0.73	0.73	0.77	0.05
0.08	accuracy	0.77	0.77	0.80	0.78	0.02
**0.07**	**accuracy**	**0.77**	**0.83**	**0.90**	**0.83**	**0.05**
0.06	accuracy	0.80	0.83	0.90	0.84	0.04

Supplementary Table 3. Influence of threshold of the variance of the tile class probability on different performance metrics.

Supplementary Figure 1

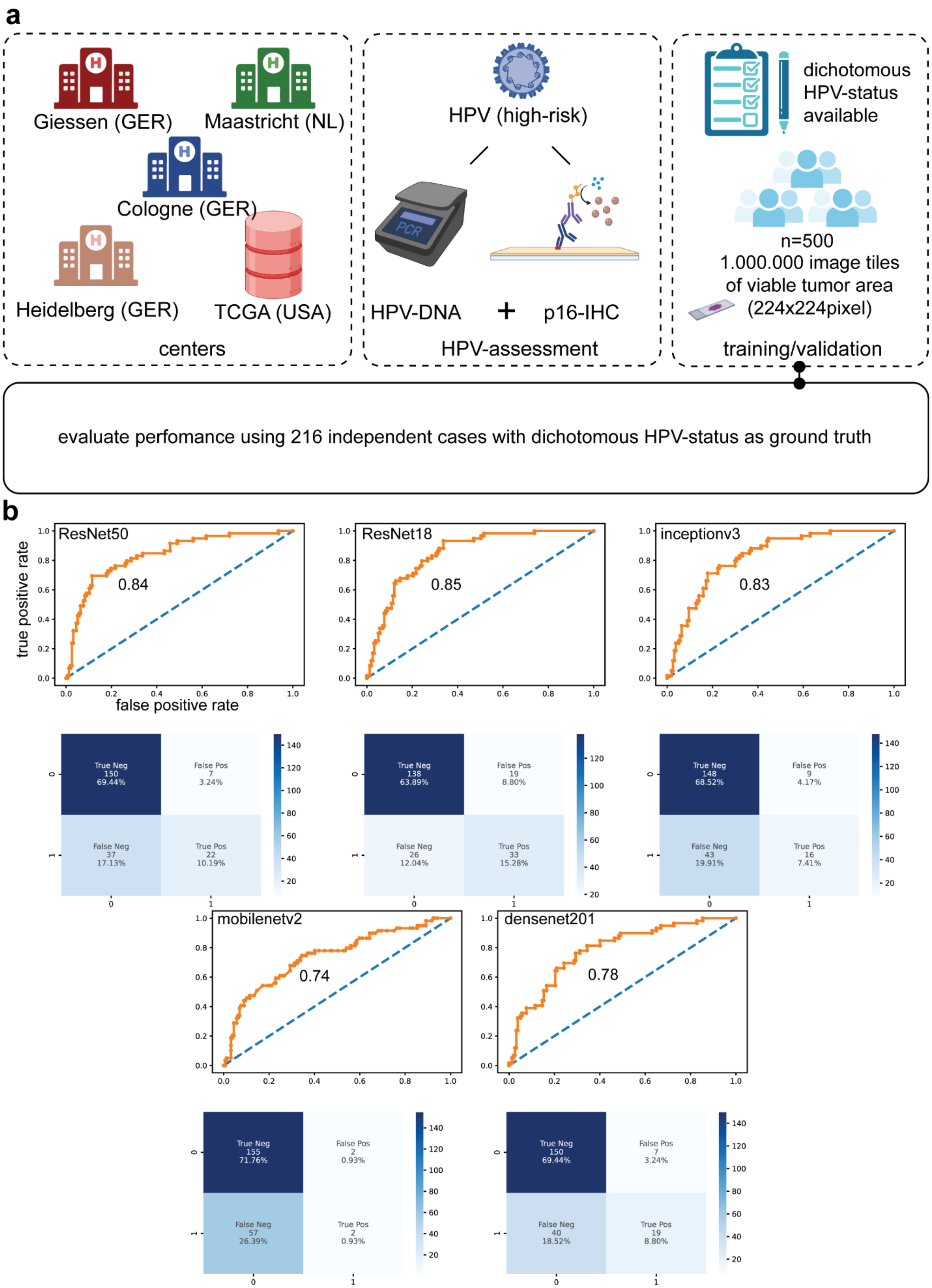

Supplementary Figure 1. Evaluating performance of five different networks for predicting HPV-association. (**a**) Schematic of the approach to identify the best performing model to predict HPV-association. Cases from four centers and one database were included in the evaluation of the best model. While the training was performed using 500 cases with 1.000.000 image tiles, there were 216 cases used for validating the algorithm. (**b**) Area under the receiver operator curve (AUROC) for the five models. In each panel the corresponding confusion matrix is visualized with true negative (True Neg), false positive (False Pos), false negative (False Neg) and true positive (True Pos) metrics being visualized.

Supplementary Figure S2

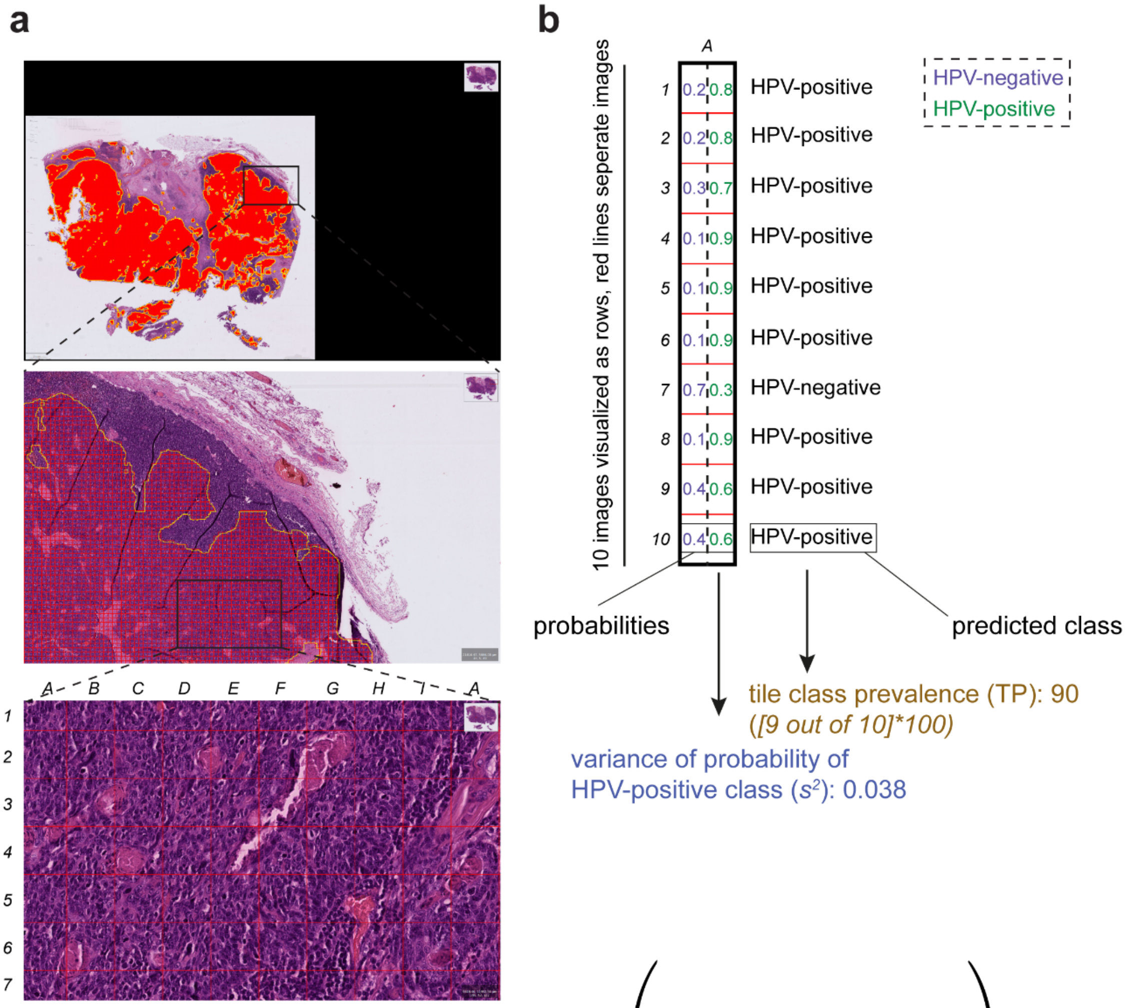

c

$$\textit{combined score} = TP * \log 2\left(\frac{1}{\left(\frac{\sum_{i=1}^{n}(x_i - \bar{X})^2}{n-1}\right)}\right)$$

Equation (3)

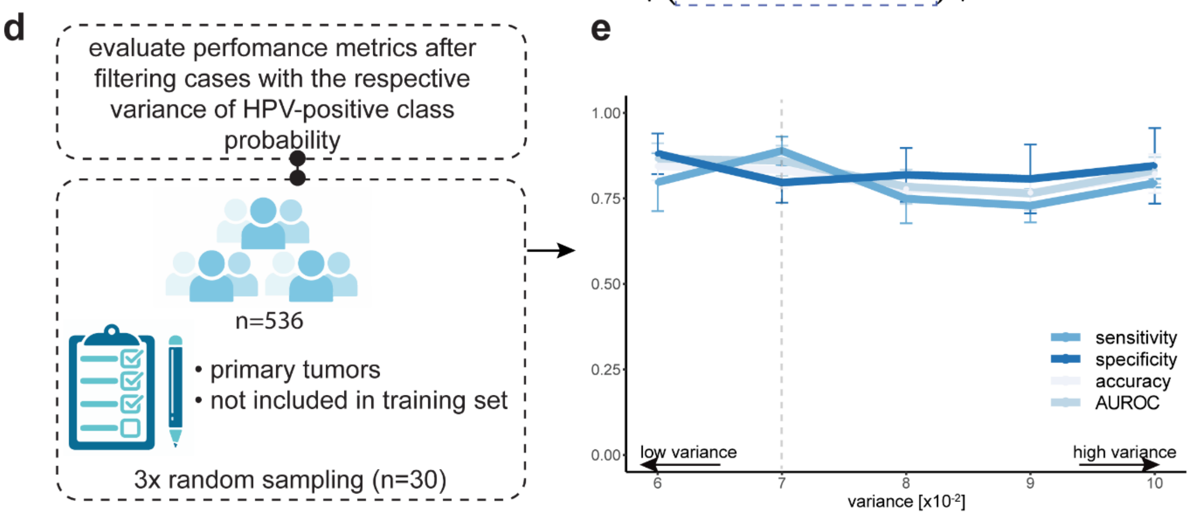

Supplementary Figure 2. Schematic explanation of scores being used for prediction of HPV-association. (**a**) One virtual whole slide image (WSI) is schematically being visualized at different magnification levels. At the highest magnification level the corresponding columns (*letters*) and rows (*numbers*) are highlighted. The grid visualized the images being tiled of the corresponding WSI. Each square has a dimension of 51.52 µm x 51.52 µm. (**b**) One column (*column A*) is shown with 10 (*row 1-10*) tiles. The numbers represent the probability which is calculated by the classification of HPV-status. The first number is the probability towards HPV-negativity, the second number is the probability towards HPV-positivity – which is color coded and explained in the legend. From the probability, the class (HPV-positive/HPV-negative) is predicted, resulting in a class for each image, in this example nine images are classified as HPV-positive, and one image is classified as HPV-negative. From the assigned classes tile class prevalence (TP) is calculated. In addition, the variance of the probability of the HPV-positive class (VAR) is calculated. Both of these are color-coded and explained in the figure. (**c**) Equation how the combined score is calculated. The color-coded boxes represent the tile class prevalence (TP) which is explained above, as well as the variance of the probability of the HPV-positive class (VAR). (**d**) Schematic of random sampling approach. The filtered cases were subsequently used for evaluating the performance of the model. (**e**) Influence on a threshold of the variance of the tile class probability of the HPV-positive class and performance metrics of the model. The error bars are illustrating the standard deviation.

Supplementary Figure 3

a

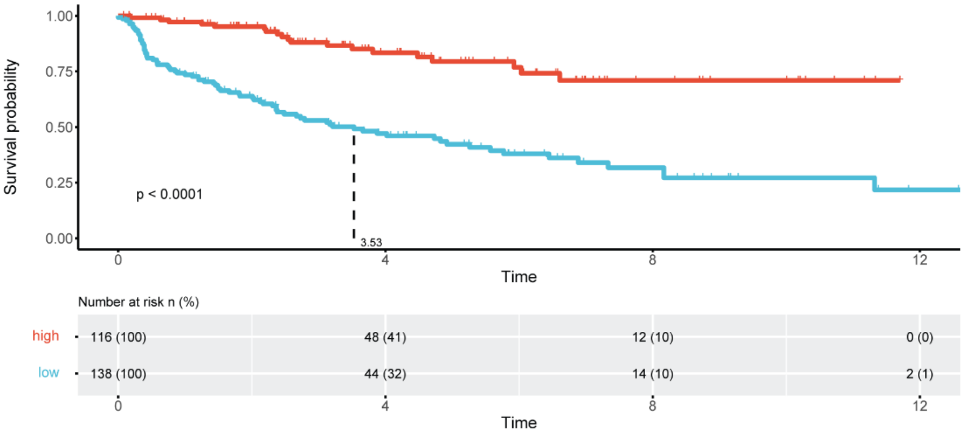

b

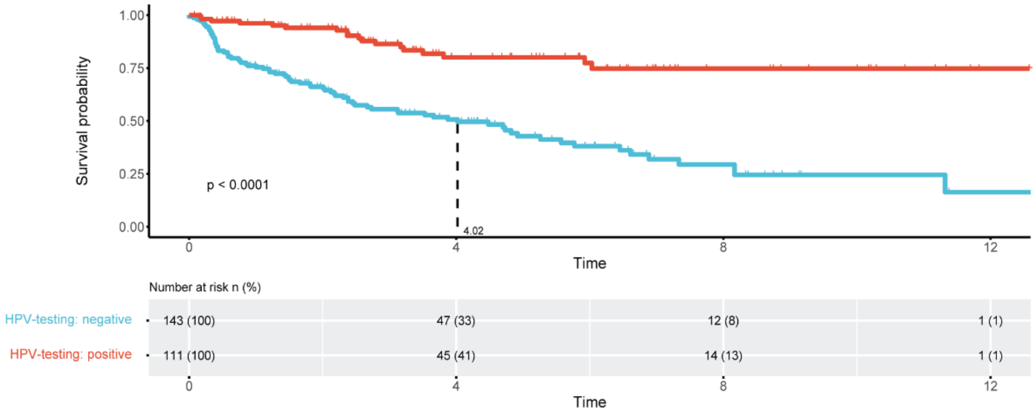

c

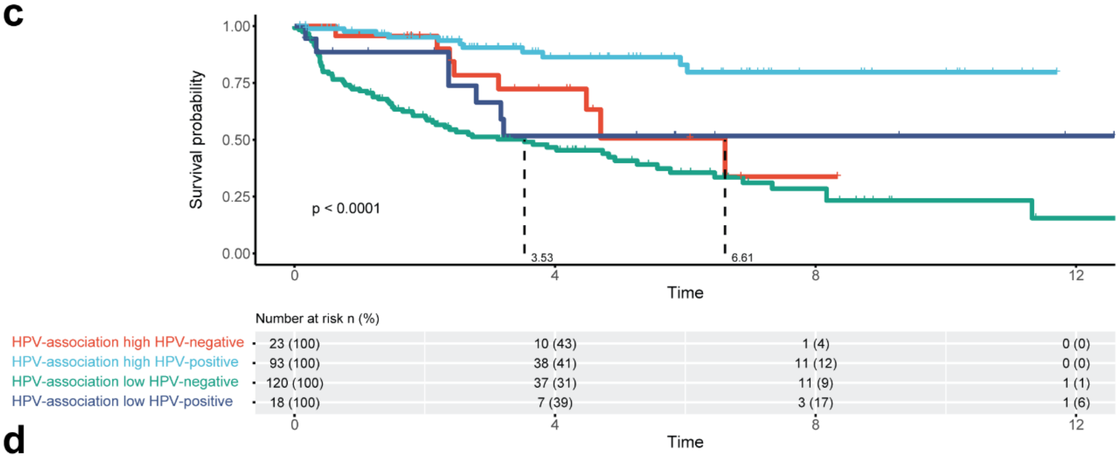

d

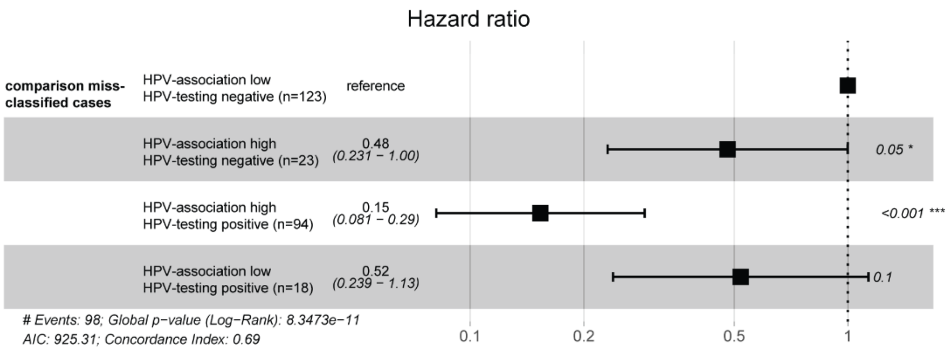

Supplementary Figure 3. Prognostic relevance of predicting HPV-association compared to regular HPV-testing and evaluation of misclassified cases. (**a**) Kaplan Meier curve for the tile class prevalence (TP) using a binary threshold of 50% to mimic a binary classification system of HPV-status (n=254). (**b**) Kaplan Meier curve for the gold standard of HPV-testing (n=254). (**c**) Kaplan Meier curve for cases with both the binary class of predicting HPV-status using OPSCCnet, as well as the ground truth of HPV-testing. Cases that were classified as HPV-positive using OPSCCnet (high) but that were negative by HPV-testing (red). Cases that were classified as HPV-positive but negative by HPV-testing (light blue). Cases that were classified as HPV-negative (low) and that were negative by HPV-testing (green). Cases that were classified as HPV-negative (low) and negative by HPV-testing (dark blue). (**d**) Forest plot of the misclassified cases from panel C (n=254). All p-values are derived by a Cox proportional hazards model.

Supplementary Figure 4

a

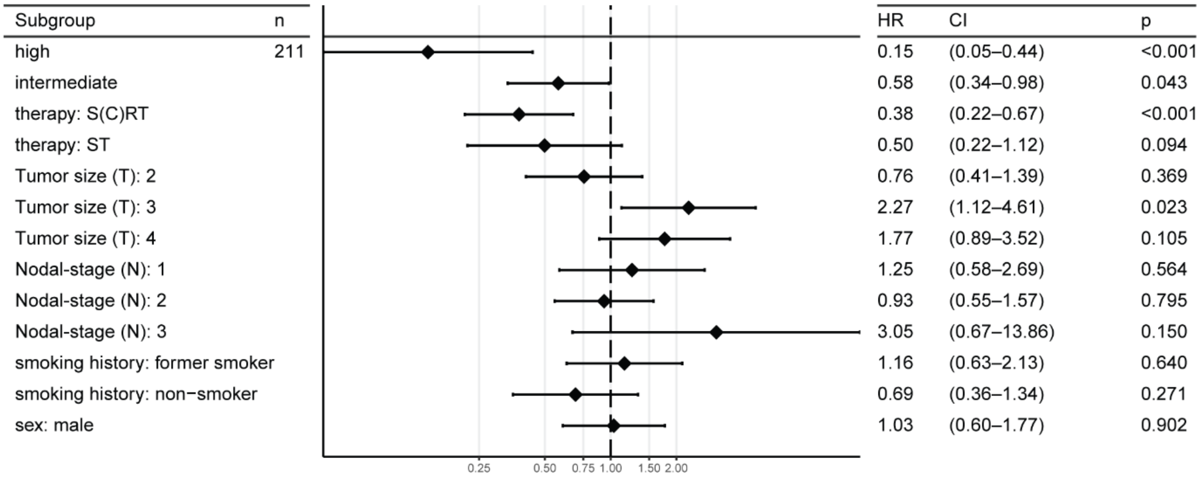

b

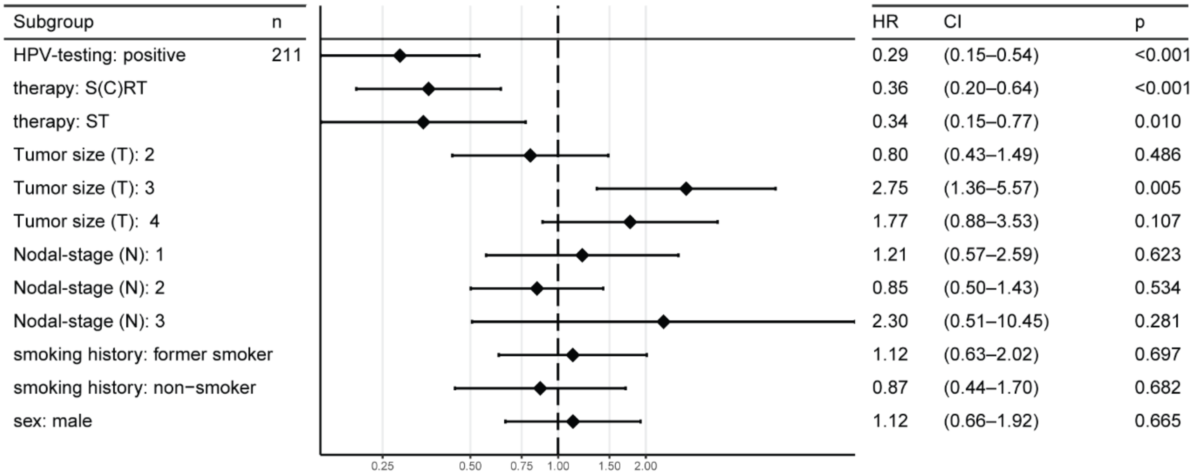

Supplementary Figure 4. Multivariate analysis of cases filtered for a certain tile class variance. (**a**) Forest plot of a multivariate analysis of cases filtered for a tile class variance below $7\ x\ 10^{-2}$. The combined score is used, and three groups are included in the analysis (high/intermediate and low: reference; n=211). (**b**) Forest plot of a multivariate analysis of cases filtered for a tile class variance below $7\ x\ 10^{-2}$ (HPV-negative: reference; same study population as A, n=211). Adjuvant radio-/chemotherapy and surgical treatment: S(C)RT; definitive radio-/chemotherapy: (C)RT; surgery alone: ST. All p-values are derived by a Cox proportional hazards model.

Supplementary Figure 5

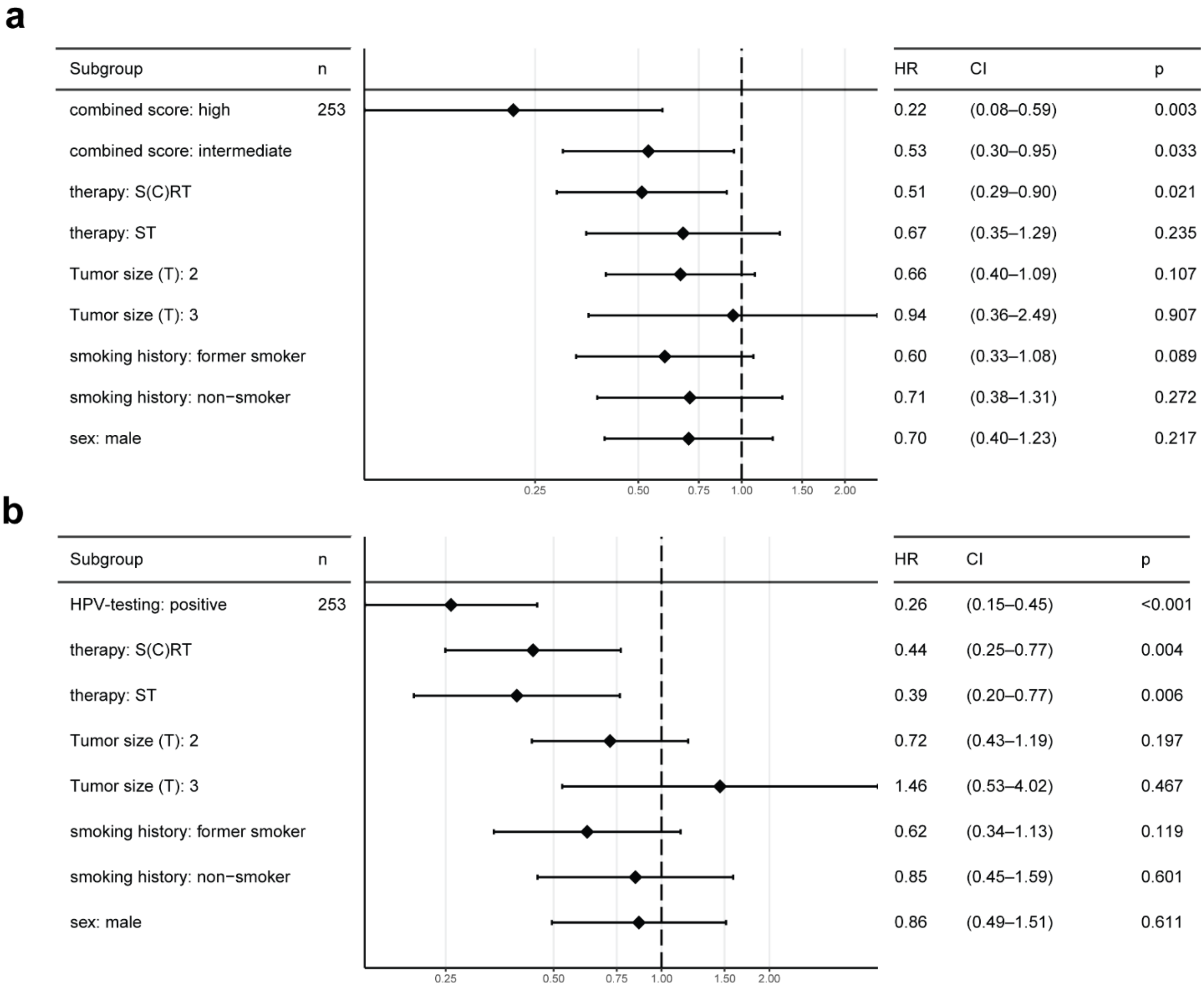

Supplementary Figure 5. Multivariate analysis of cases filtered for stage I/II disease. (**a**) Forest plot of a multivariate analysis using the combined score (high/intermediate and low: reference; n=253). (**b**) Forest plot of the survival for regular HPV-testing (HPV-negative: reference) and several clinical variables. Same study population as A (n=253). Adjuvant radio-/chemotherapy and surgical treatment: S(C)RT; definitive radio-/chemotherapy: (C)RT; surgery alone: ST. All p-values are derived by a Cox proportional hazards model.

Supplementary Figure 6

a

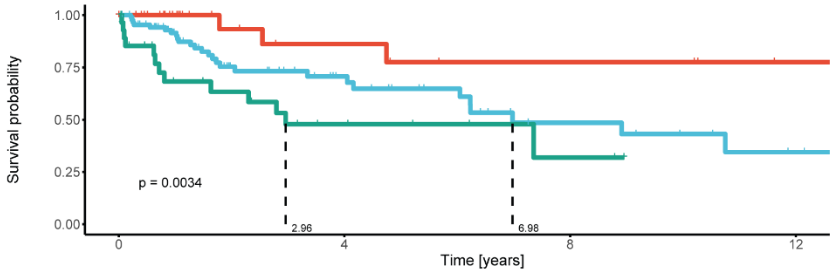

Number at risk n (%)

	0	4	8	12
combined score=high	31 (100)	10 (32)	7 (23)	4 (13)
combined score=intermediate	90 (100)	24 (27)	9 (10)	3 (3)
combined score=low	30 (100)	6 (20)	2 (7)	0 (0)

Time [years]

b

Subgroup	n	HR	CI	p
combined score: high	136	0.10	(0.02–0.50)	0.005
combined score: intermediate		0.43	(0.16–1.14)	0.091
therapy:S(C)RT		0.33	(0.12–0.89)	0.028
therapy :ST		0.42	(0.14–1.24)	0.117
tumor size (T): 2		1.76	(0.75–4.13)	0.193
tumor size (T): 3		4.17	(0.52–33.41)	0.179
smoking history: former smoker		0.83	(0.36–1.89)	0.654
smoking history: non−smoker		0.94	(0.41–2.17)	0.879
sex:male		0.80	(0.37–1.72)	0.563

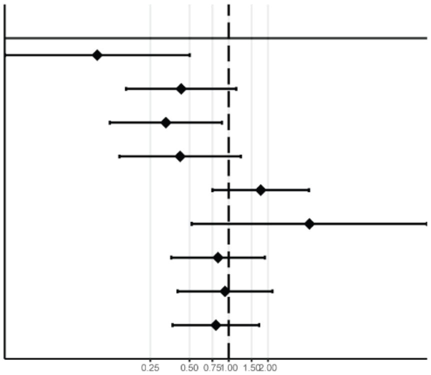

c

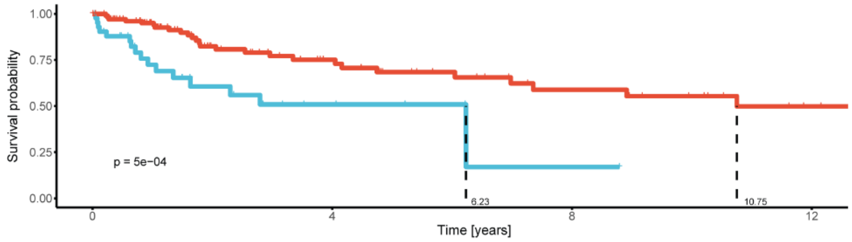

Number at risk n (%)

	0	4	8	12
HPV-negative	45 (100)	6 (13)	1 (2)	0 (0)
HPV-positive	106 (100)	34 (32)	17 (16)	7 (7)

Time [years]

D

Subgroup	n	HR	CI	p
HPV-testing: positive	136	0.22	(0.09–0.53)	<0.001
therapy: S(C)RT		0.27	(0.10–0.74)	0.011
therapy: ST		0.30	(0.11–0.80)	0.016
tumor size (T): 2		2.20	(0.94–5.16)	0.070
tumor size (T): 3		5.19	(0.62–43.51)	0.129
smoking history: former smoker		0.76	(0.34–1.71)	0.510
smoking history: non−smoker		0.94	(0.41–2.12)	0.874
sex:male		0.69	(0.32–1.50)	0.352

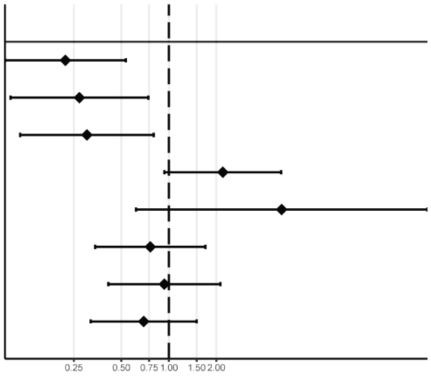

Supplementary Figure 6. Stratification of the training cohort using the combined score. (**a**) Kaplan-Meier plot for patients with stage I/II disease are stratified using the combined score (n=151). (**b**) Forest plot of a multivariate analysis of patients with stage I/II disease stratified for the combined score and several clinical variables (n=136). (**c**) Kaplan-Meier plot for patients with stage I/II disease stratified for regular HPV-testing (n=151, same population as A). (**d**) Forest plot of a multivariate analysis for patients with stage I/II disease, stratified for regular HPV-testing (HPV-negative: reference, n=136, same study population as B). All p-values are derived by a Cox proportional hazards model.

Supplementary Figure 7

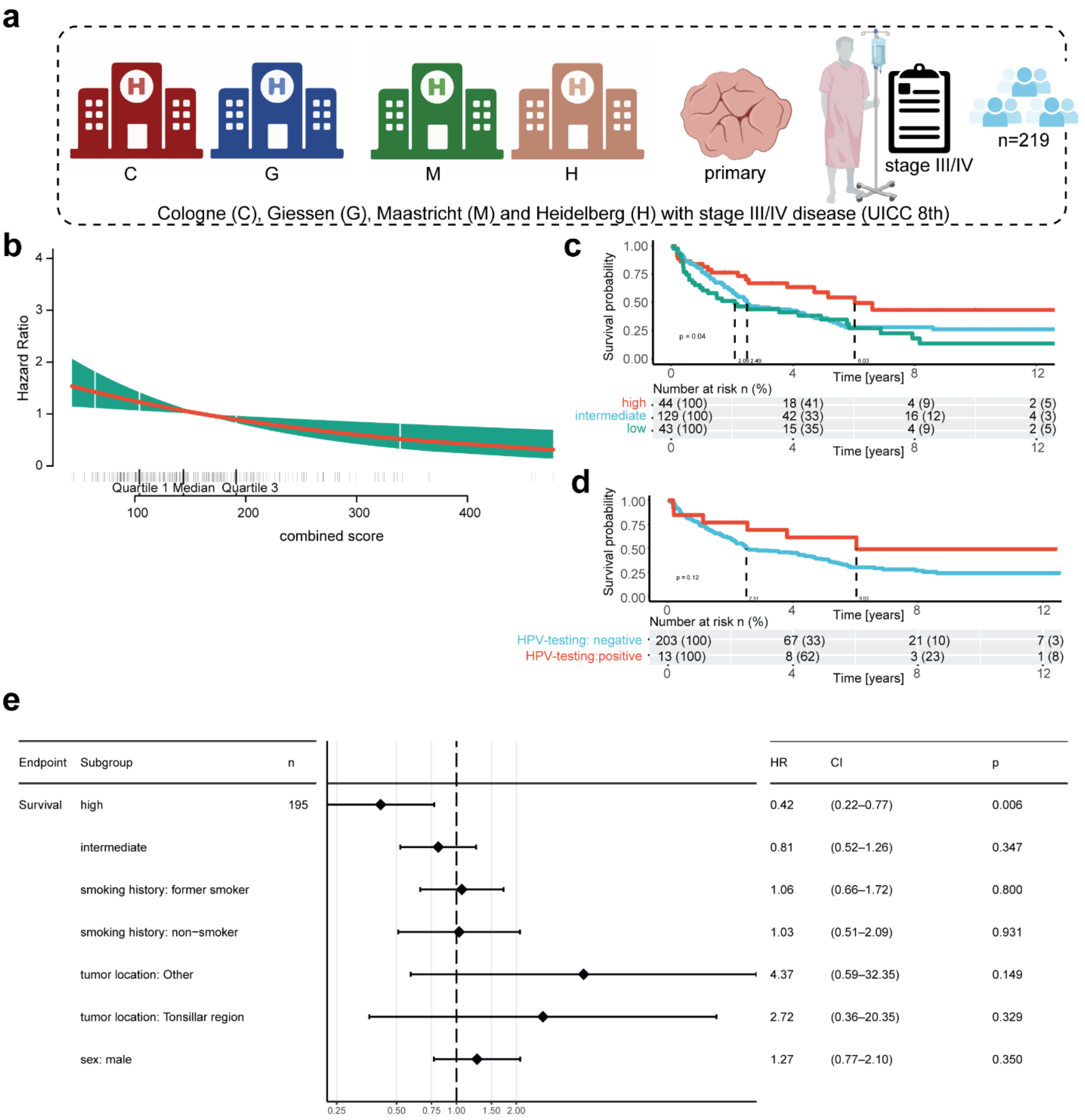

Supplementary Figure 7. Stratification of patients with advanced stage disease (**a**) Schematic illustration of the filtering criteria for the subsequent analysis. Cases from Cologne, Giessen, Maastricht, and Heidelberg with stage III/IV disease are included for the following analysis (n=219) (**b**) Hazard ratio plot of the selected patients with stage III/IV disease using the combined score. The red line indicates the smoothened function,

the vertical lines at the horizontal axis indicates individual patients with a given risk. The green bar indicates the error of the function (**c**) Kaplan-Meier curve for the combined score (n=219), divided into three groups (high/intermediate/low) (**d**) Kaplan-Meier curve for regular HPV-testing, divided into HPV-positive and HPV-negative (n=219). (**e**) Multivariate analysis of patients with a positive HPV-status stratified for the combined score (n=195). All p-values are derived by a Cox proportional hazards model.

Supplementary Figure 8

Subgroup	n	HR	CI	p
high	196	0.21	(0.05–0.87)	0.032
intermediate		0.54	(0.24–1.20)	0.128
therapy: S(C)RT		0.52	(0.23–1.16)	0.112
therapy: ST		1.74	(0.45–6.62)	0.420
tumor location: Other		0.32	(0.07–1.47)	0.142
tumor location: Tonsillar region		0.33	(0.08–1.36)	0.126
Tumor size (T): 2		0.61	(0.25–1.49)	0.274
Tumor size (T): 3		2.03	(0.62–6.66)	0.245
Tumor size (T): 4		3.75	(1.21–11.59)	0.022
Nodal-stage (N): 1		1.32	(0.32–5.46)	0.704
Nodal-stage (N): 2		2.31	(0.77–6.94)	0.136
Nodal-stage (N): 3		5.27	(0.80–34.74)	0.084
smoking history: former smoker		1.28	(0.53–3.07)	0.586
smoking history: non−smoker		0.85	(0.35–2.05)	0.719
sex: male		0.56	(0.22–1.37)	0.202

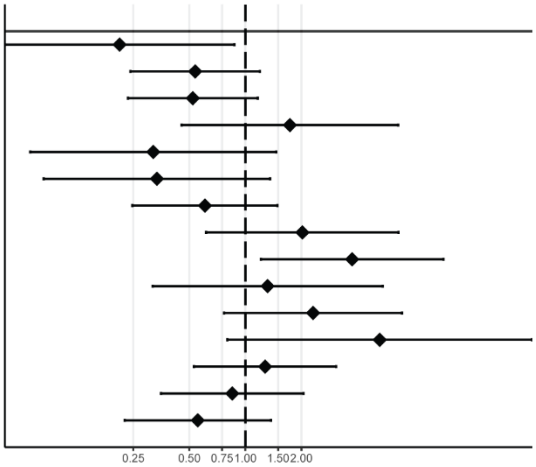

Supplementary Figure 8. Multivariate analysis of patients with a positive HPV-status. Multivariate analysis of patients with a positive HPV-status stratified for the combined score. Adjuvant radio-/chemotherapy and surgical treatment: S(C)RT; definitive radio-/chemotherapy: (C)RT; surgery alone: ST. All p-values are derived by a Cox proportional hazards model.

Supplementary Figure 9

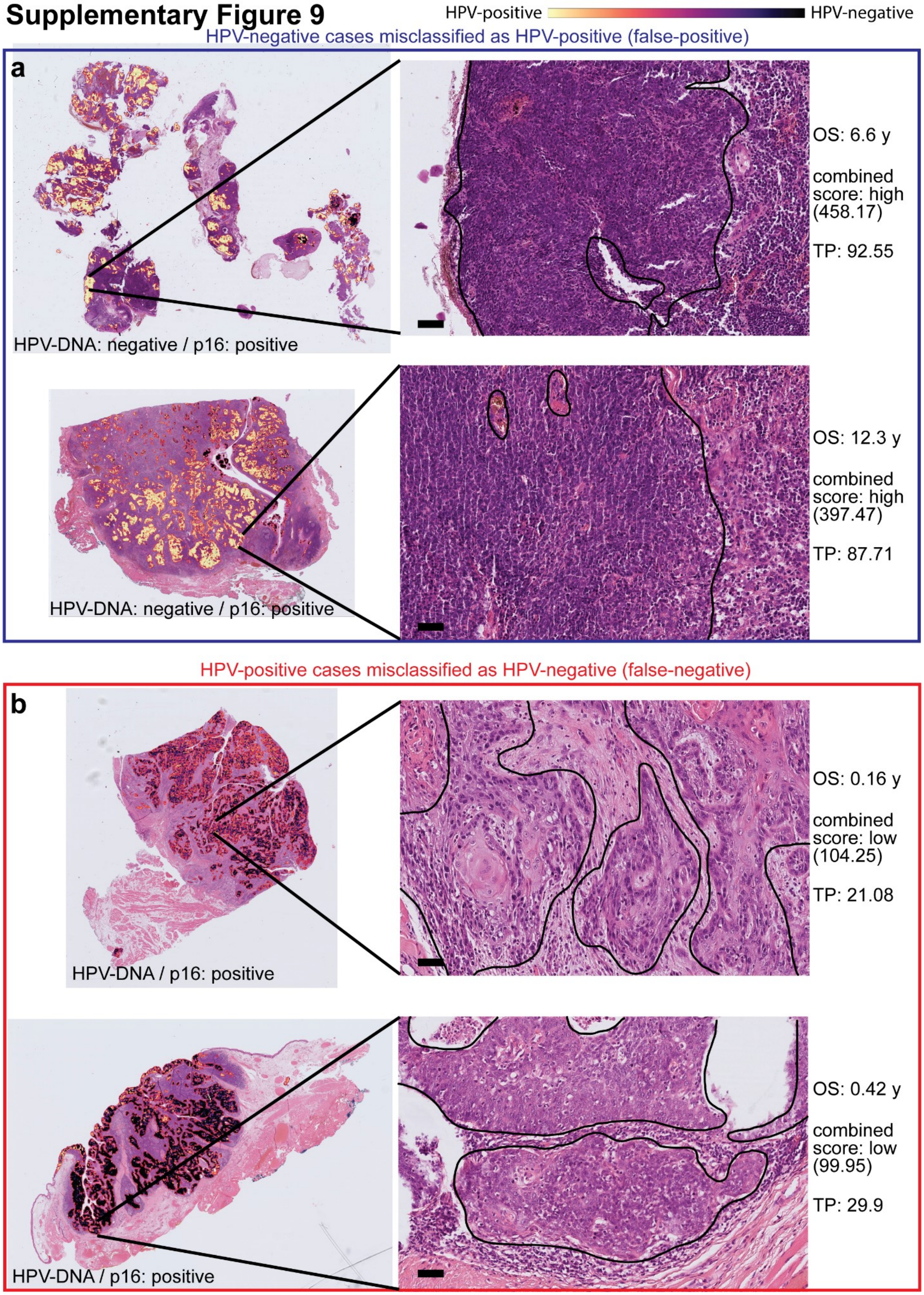

Supplementary Figure 9. Visualization of misclassified cases using both prediction maps and high-resolution tiles. (**a**) HPV-negative cases that have been falsely classified as HPV-positive are visualized. The left panel shows the whole-slide prediction map with a yellow color code for showing areas with high probability of HPV-positivity. The right panel shows a high-resolution image of the respective tumor with tumor areas circled. The overall survival in years (OS; y), together with the tile class prevalence (TP) is shown on the right side. (**b**) HPV-positive cases that have been falsely classified as HPV-negative are visualized. The left panel shows the whole-slide prediction map with a black color code for showing areas with high probability of HPV-negativity. The right panel shows a high-resolution image of the respective tumor with tumor areas circled. The overall survival in years (OS; y), together with the tile class prevalence (TP) is shown on the right side. Scale bars are 50 μm each.
