# Supplementary material for: Predicting HPV association using deep learning and regular H&E stains allows granular stratification of oropharyngeal cancer patients
Source: NPJ Digit Med. 2023 Aug 19;6:152. doi: 10.1038/s41746-023-00901-z (PMC10439941; doi:10.1038/s41746-023-00901-z)
Supplement: Supplementary file 2 — Reporting Summary [file 41746_2023_901_MOESM2_ESM.pdf]

nature portfolio

Corresponding author(s): Sebastian Klein (MD)

Last updated by author(s): Feb 14, 2023

Reporting Summary

Nature Portfolio wishes to improve the reproducibility of the work that we publish. This form provides structure for consistency and transparency in reporting. For further information on Nature Portfolio policies, see our Editorial Policies and the Editorial Policy Checklist.

Statistics

For all statistical analyses, confirm that the following items are present in the figure legend, table legend, main text, or Methods section.

n/a	Confirmed	
☐	☒	The exact sample size (*n*) for each experimental group/condition, given as a discrete number and unit of measurement
☐	☒	A statement on whether measurements were taken from distinct samples or whether the same sample was measured repeatedly
☐	☒	The statistical test(s) used AND whether they are one- or two-sided<br>*Only common tests should be described solely by name; describe more complex techniques in the Methods section.*
☐	☒	A description of all covariates tested
☐	☒	A description of any assumptions or corrections, such as tests of normality and adjustment for multiple comparisons
☐	☒	A full description of the statistical parameters including central tendency (e.g. means) or other basic estimates (e.g. regression coefficient) AND variation (e.g. standard deviation) or associated estimates of uncertainty (e.g. confidence intervals)
☐	☒	For null hypothesis testing, the test statistic (e.g. *F*, *t*, *r*) with confidence intervals, effect sizes, degrees of freedom and *P* value noted<br>*Give P values as exact values whenever suitable.*
☒	☐	For Bayesian analysis, information on the choice of priors and Markov chain Monte Carlo settings
☒	☐	For hierarchical and complex designs, identification of the appropriate level for tests and full reporting of outcomes
☐	☒	Estimates of effect sizes (e.g. Cohen's *d*, Pearson's *r*), indicating how they were calculated

Our web collection on statistics for biologists contains articles on many of the points above.

Software and code

Policy information about availability of computer code

Data collection	Python 3.8, R 4.1 / R-Studio 1.4
Data analysis	Python 3.8, R 4.1 / R-Studio 1.4<br>https://github.com/OPSCCnet/OPSCCnet

For manuscripts utilizing custom algorithms or software that are central to the research but not yet described in published literature, software must be made available to editors and reviewers. We strongly encourage code deposition in a community repository (e.g. GitHub). See the Nature Portfolio guidelines for submitting code & software for further information.

Data

Policy information about availability of data

All manuscripts must include a data availability statement. This statement should provide the following information, where applicable:

- Accession codes, unique identifiers, or web links for publicly available datasets
- A description of any restrictions on data availability
- For clinical datasets or third party data, please ensure that the statement adheres to our policy

The datasets from the four centers cannot be made publicly available because of privacy requirements of the participating medical centers.

Human research participants

Policy information about studies involving human research participants and Sex and Gender in Research.

Reporting on sex and gender	Throughout this study, we exclusively report on biological Sex (using multivariate analysis). The information on Gender has not been collected for this study population.
Population characteristics	906 patients with OPSCCs from four centers and one database were enrolled in this retrospective study. All patients were treated in accordance with approved guidelines by either surgery alone (ST) or upfront surgery and concomitant (chemo)radiotherapy (S(C)RT) or definitive chemoradiotherapy ((C)RT). Overall survival was defined as the time after initial diagnosis to death from any cause. A detailed description of the patient population characteristics can be found in Table S1.
Recruitment	Patients from Cologne, Giessen, Heidelberg, and Maastricht diagnosed with primary squamous cell carcinoma of the oropharynx (ICD code C10, International Classification of Diseases for Oncology) and treated at the given local center between 2005 and 2019).
Ethics oversight	The study was conducted in accordance with the Declaration of Helsinki, and the protocol was approved by the regional ethics committees (Giessen: AZ 95/15, dated October 19, 2015; Cologne: AZ 19–1288_1, dated February 3, 2020). Informed written consent was obtained from each subject. Patient characteristics were recorded prospectively by the Giessen cancer registry database (GTDS), as well as from the cancer registry database of the Center for Integrated Oncology (CIO), Cologne. H&E stained tumor samples were provided by the Tissue Bank of the National Center for Tumor Diseases (NCT) Heidelberg, Germany in accordance with the regulations of the tissue bank and the approval of the ethics committee of Heidelberg University (S-207/2005 and S-786/2021).17 Ethical approval for use of the Maastricht samples was granted by the local ethical committee under the study number METC-2021 2658.

Note that full information on the approval of the study protocol must also be provided in the manuscript.

Field-specific reporting

Please select the one below that is the best fit for your research. If you are not sure, read the appropriate sections before making your selection.

☒ Life sciences ☐ Behavioural & social sciences ☐ Ecological, evolutionary & environmental sciences

For a reference copy of the document with all sections, see nature.com/documents/nr-reporting-summary-flat.pdf

Life sciences study design

All studies must disclose on these points even when the disclosure is negative.

Sample size	Sample size was determined by the availability of appropriate tissue material and clinical data. No statistical method was used for sample size estimation or calculation. Globally, we aimed to include as many patients [retrospective data] as possible.
Data exclusions	Data inclusion and exclusion criteria were determined before initiation of the retrospective study. Patients from Cologne, Giessen, Heidelberg, and Maastricht diagnosed with primary squamous cell carcinoma of the oropharynx (ICD code C10, International Classification of Diseases for Oncology) and treated at the given local center between 2005 and 2019). CONSORT diagrams of the cases used for training, validation and testing can be found in Figure 1. Briefly cases with wrong anatomical localization of the tumor have been excluded, or patients with missing information on HPV-status. Patients with follow-up of 0 years and lost to follow-up were excluded from the survival analysis.
Replication	By making our algorithm and the weights publicly available (OPSCCnet), we aim to make our results reproducible. Our approach of using quantifiable measures of prognosis (Hazard-ratio, Likelihood-Ratio test) using cox-proportional hazard models is more informative than previous studies comparing solely ranked based prognostic markers (including concordance-index). We also reveal quantifiable prognostic measures, including a five-year survival rate, which are comparable to other studies.
Randomization	By following a random sub-sampling approach (Figure S2D,E), we show that our results within the external test-set are unbiased. Given a large external test-set of different centers and even origin of the tumor (lymph-node metastases) we show generalizability of our model.
Blinding	No blinding was used in our experiments. As it was a retrospective study, all relevant data had been collected prior to training, validation, and testing of the model, so there was no need/way to perform this in a blinded fashion. No subjective evaluation which required blinding was performed in our study.

Reporting for specific materials, systems and methods

We require information from authors about some types of materials, experimental systems and methods used in many studies. Here, indicate whether each material, system or method listed is relevant to your study. If you are not sure if a list item applies to your research, read the appropriate section before selecting a response.

Materials & experimental systems

n/a	Involved in the study
☒	☐ Antibodies
☒	☐ Eukaryotic cell lines
☒	☐ Palaeontology and archaeology
☒	☐ Animals and other organisms
☒	☐ Clinical data
☒	☐ Dual use research of concern

Methods

n/a	Involved in the study
☒	☐ ChIP-seq
☒	☐ Flow cytometry
☒	☐ MRI-based neuroimaging
